# Comparative efficacy and safety of six traditional Chinese medicine injections for ischemic heart failure: a systematic review and Bayesian network meta-analysis

**DOI:** 10.3389/fcvm.2026.1969858

**Published:** 2026-10-30

**Authors:** Yu Shang, Penglu Wei, Yue Mao, Jinhao Tian, Xiaolei Lai, Wenlong Xing, Juju Shang, Hongxu Liu

**Affiliations:** 1 Graduate School, Beijing University of Chinese Medicine, Beijing, China; 2 Beijing Hospital of Traditional Chinese Medicine, Capital Medical University, Beijing, China

**Keywords:** ischemic heart failure, network meta-analysis, randomized controlled trial, systematic review, traditional Chinese medicine injection

## Abstract

**Background:**

Traditional Chinese medicine injections (TCMIs) are widely employed by Chinese clinicians as adjuvant therapy for Ischemic Heart Failure(IHF). A Bayesian network meta-analysis was conducted to compare the efficacy and safety of conventional treatment (CT) to those that included six TCMIs in patients with IHF.

**Objective:**

To compare the efficacy and safety of six TCMIs as adjuncts to CT for IHF using Bayesian network meta-analysis.

**Methods:**

Eight databases (CNKI, Wanfang, VIP, SinoMed, PubMed, Embase, the Cochrane Library, and Web of Science) were searched from inception to May 6, 2026, for randomized controlled trials (RCTs). Risk of bias was assessed using RoB 2.0, certainty of evidence using CINeMA, and Bayesian analyses using R 4.6.0 and Stata 17.0.

**Results:**

A total of 154 RCTs involving 13,756 participants were included. Compared with CT alone, adjunctive TCMIs generally improved clinical response and left ventricular ejection fraction(LVEF), and reduced B-type natriuretic peptide(BNP) and left ventricular end-diastolic dimension(LVEDD); effects on N-terminal pro-B-type natriuretic peptide(NT-pro BNP) were uncertain. Surface under the cumulative ranking curve(SUCRA) estimates Shenmai injection(SMI) had the highest probability of ranking favorably for clinical effectiveness rate(CER) and left ventricular ejection fraction(LVEF), Shengmai injection(SHMI) for B-type natriuretic peptide(BNP), Xinmailong injection(XMLI) for N-terminal pro-B-type natriuretic peptide(NT-pro BNP), Yiqifumai injection(YQFMI) for left ventricular end-diastolic dimension(LVEDD), and Shenfu injection(SFI) for 6-minute walking distance(6MWT). Short-term adverse-event rates were not increased overall.

**Conclusion:**

TCMIs combined with CT has potential benefits in the treatment of IHF, but confidence in treatment rankings is limited by low or very low certainty of evidence, substantial heterogeneity for several outcomes, methodological limitations, and the absence of direct head-to-head trials. Large, rigorously designed RCTs with long-term safety follow-up are required.

**Systematic Review Registration:**

https://www.crd.york.ac.uk/PROSPERO/, identifier (CRD 420261380716).

## 1 Introduction

Heart failure (HF) is a clinical syndrome characterized by abnormalities in cardiac structure and/or function, leading to impaired ventricular systolic and/or diastolic function (1). It represents the end-stage of various cardiac diseases, and its high incidence, mortality, and hospitalization rates—coupled with poor quality of life—impose a heavy socioeconomic burden (2–8). HF has become a major global public health concern, with its prevalence continuing to rise. Epidemiological data indicate that approximately 56 million people worldwide live with HF (1), including around 12.1 million adults in China (9). This upward trend is projected to persist until 2050, driven by population aging, improved survival rates in patients with coronary atherosclerotic heart disease (CHD), and better outcomes in acute cardiovascular event care (10).

Ischemic heart disease (IHD), a condition caused by coronary artery stenosis or occlusion due to atherosclerosis, leads to myocardial ischemia, hypoxia, and even necrosis (11, 12). It is one of the most common etiologies of HF. A multicenter study including over 43,000 HF patients from 24 countries and regions found that coronary artery disease was the underlying cause in 62% of HF cases (13). As the end-stage of IHD, ischemic heart failure (IHF) is marked by key pathological changes including cardiomyocyte apoptosis, myocardial fibrosis, and ventricular remodeling—all triggered by persistent or recurrent myocardial ischemia. Its underlying pathological mechanisms involve endothelial injury, inflammatory responses, oxidative stress, and neuroendocrine activation. In recent years, guideline-directed medical therapy (GDMT) regimens have been continuously refined. The widespread use of medications such as angiotensin receptor-neprilysin inhibitors (ARNIs)/angiotensin-converting enzyme inhibitors (ACEIs)/angiotensin II receptor blockers (ARBs), *β*-blockers, mineralocorticoid receptor antagonists (MRAs), sodium-glucose cotransporter 2 inhibitors (SGLT2is), and soluble guanylate cyclase (sGC) stimulators has reduced the risk of cardiovascular death and rehospitalization due to worsening HF. Compared with non-ischemic HF, IHF patients usually present with more severe coronary artery lesions, requiring a multi-target integrated treatment strategy.

In traditional Chinese medicine (TCM), IHF falls under the categories of “heart failure”, “heart water”, and “edema”. TCM has accumulated rich experience in HF prevention and treatment, and integrated Chinese-Western medicine has shown confirmed efficacy in clinical IHF management. In recent years, traditional Chinese medicine injections (TCMIs) have gradually emerged as an adjuvant therapy for IHF patients, thanks to their multi-component, multi-target synergistic effects, high bioavailability, and rapid onset of action. A growing number of clinical studies and systematic reviews have confirmed TCMI efficacy in IHF, yet the comparative efficacy and safety of different TCMIs remain to be further clarified.

Network meta-analysis (NMA) is a systematic review method that constructs an evidence network covering multiple treatment options, allowing simultaneous comparison of direct and indirect evidence across multiple regimens to deliver more comprehensive and precise support for clinical decision-making (14). Based on the Guidelines for TCM Diagnosis and Treatment of Chronic Heart Failure (2022) (15), this study selected six TCMIs recommended for HF treatment: Shengmai injection (SHMI), Shenmai injection (SMI), Shenfu injection (SFI), Xinmailong injection (XMLI), Huangqi injection (HQI), and Yiqifumai injection (YQFMI). We used Bayesian NMA to systematically compare the efficacy and safety of the six TCMIs as add-on therapy to conventional treatment (CT) in IHF patients, and evaluated evidence quality using the CINeMA framework. The study aims to provide evidence-based support for precise TCMI use in IHF management and help optimize treatment strategies.

## 2 Methods

This study was conducted in accordance with the PRISMA-NMA statement as seen in Supplementary Table S1, and registered on the PROSPERO platform (registration number: CRD420261380716).

### 2.1 Literature retrieval

We performed electronic searches for published literature across eight Chinese and English databases: China National Knowledge Infrastructure (CNKI), WanFang Academic Journals Database (Wanfang), VIP Chinese Scientific Journals Database (VIP), Chinese Biomedical Literature Service System (SinoMed), PubMed, EMbase, the Cochrane Library, and Web of Science, with no language restrictions. The search covered the period from database inception to May 6, 2026. A combination of Medical Subject Headings (MeSH) and free-text terms was used for searching. Chinese search terms included ischemic heart disease, ischemic heart failure, coronary heart disease, coronary atherosclerotic heart disease, heart failure, cardiac insufficiency, and randomization. Search strategies for the network meta-analysis are in Supplementary Table S2.

### 2.2 Inclusion and exclusion criteria

#### 2.2.1 Inclusion criteria

##### 2.2.1.1 Study participants

Patients with a confirmed IHF diagnosis (including HF due to coronary heart disease and post-myocardial infarction HF) per relevant diagnostic criteria were included, with no restrictions on sex, age, disease duration, ethnicity, or geographic region.

##### 2.2.1.2 Interventions

The control group received CT, including ARNIs, ACEIs, ARBs, *β*-blockers, diuretics, MRAs, SGLT2is, antiplatelet drugs, nitrates, statins, and digitalis preparations. The experimental group received one of the six TCMIs in addition to CT. Injection dosage strictly followed drug instructions, and intervention duration was no less than 7 days.

##### 2.2.1.3 Outcomes included

(1) Clinical effectiveness rate (CER), defined as: ①Markedly effective: Symptoms and signs disappear, HF is controlled, and cardiac function improves by ≥2 grades or returns to grade 1; ②Effective: Symptoms and signs are significantly relieved, and cardiac function improves by 1 grade but less than 2 grades; ③Ineffective: No significant improvement or worsening of symptoms and signs, with cardiac function improvement of less than 1 grade or even deterioration. Total effective rate = (number of markedly effective cases + number of effective cases)/sample size  ×  100%. (2) Adverse drug reactions (ADRs); (3) Left ventricular ejection fraction (LVEF); (4) Left ventricular end-diastolic dimension (LVEDD); (5) B-type natriuretic peptide (BNP); (6) N-terminal pro-brain natriuretic peptide (NT-proBNP); (7) 6-minute walk test (6MWT).

##### 2.2.1.4 Study type

Randomized controlled trials (RCTs).

#### 2.2.2 Exclusion criteria

(1) Studies where HF caused by other etiologies could not be ruled out based on study content; (2) Duplicate publications; (3) Case reports, reviews, literature studies, theoretical discussions, personal experience summaries, and non-RCT studies; (4) Studies where the control group received other TCM or TCM-based treatments, with unclear dosage or treatment duration; (5) Studies with too few reported cases per group (<20 cases per group); (6) Inaccurate or incomplete data; (7) Full text unavailable.

### 2.3 Literature screening and data extraction

Two researchers independently conducted literature screening and data extraction. Retrieved bibliographic records were imported into EndNote 20 for initial screening and deduplication. Irrelevant literature was excluded by reading titles and abstracts, and remaining studies were assessed against inclusion/exclusion criteria. Disagreements between the two researchers were resolved through discussion with a third researcher to finalize included RCTs. Microsoft Excel 2017 was used for data extraction and entry. Extracted information included first author, publication year, sample size, mean age, disease duration, interventions, intervention duration, follow-up time, outcome measures, and literature quality assessment indicators. When outcome measures were measured at multiple time points, only data from the last time point were included.

### 2.4 Risk of bias assessment and evidence quality evaluation

The Cochrane Risk of Bias tool (RoB 2.0) was used to assess risk of bias in included studies, covering six domains: bias arising from the randomization process, bias due to deviations from intended interventions, bias due to missing outcome data, bias in outcome measurement, bias in selection of reported results, and other biases. The CINeMA online tool (https://cinema.ispm.unibe.ch/#) was applied to evaluate evidence quality for included studies. CINeMA covers six domains: within-study bias, between-study bias, indirectness, imprecision, heterogeneity, and incoherence. Two researchers independently assessed risk of bias and evidence quality, with disagreements resolved through discussion or consultation with a third researcher.

### 2.5 Data statistics and analysis

Bayesian network meta-analysis was performed using R (version 4.6.0) and Stata (version 17.0), with simulations conducted via the Markov Chain Monte Carlo (MCMC) method. We set 50,000 sample iterations, including 20,000 burn-in iterations, with a step size of 1, until model convergence. Since no head-to-head RCTs were available for this study and all data came from indirect comparisons—with no closed loops in the network structure, a consistency model was used for both network meta-analysis and treatment ranking analysis. For dichotomous variables, the odds ratio (*OR*) with 95% confidence interval (*CI*) was used as the effect statistic. For continuous variables, BNP and NT-proBNP were expressed as standardized mean differences (*SMD*) with 95% *CI*, while LVEF, LVEDD, and 6MWT were expressed as mean differences (*MD*) with 95% *CI*. When SDs of change scores were not reported, they were imputed from baseline and post-intervention SDs using an assumed within-study correlation coefficient, following the Cochrane Handbook. The primary analysis used *r* = 0.5 for BNP/NT-proBNP (16), *r* = 0.8 for LVEF/LVEDD (17), and *r* = 0.7 for 6MWT (18). Because these correlations were not consistently reported in the included original trials, we conducted prespecified sensitivity analyses using lower and higher plausible correlation coefficients to evaluate the robustness of the imputation assumption. A difference was considered statistically significant when the 95% *CI* of *OR* did not include 1, or the 95% *CI* of *SMD/MD* did not include 0. For outcomes involving more than 10 RCTs, funnel plots were generated to assess publication bias potential. Surface under the cumulative ranking curve(SUCRA) values (ranging from 0 to 100%) were used to rank the efficacy and safety of different interventions, with higher SUCRA values indicating a better ranking. The global *I*^2^ statistic and prediction interval plots were used to assess heterogeneity degree, with an *I*^2^ value exceeding 50% considered high heterogeneity. Regardless of whether significant heterogeneity was detected, a random-effects model was used for meta-analysis to ensure consistency across indicators. To verify result robustness, sensitivity analysis was performed on studies with ≥100 cases. Subgroup analyses were conducted based on TCMI dosage, treatment duration, age of participants, and IHF classification. In addition, conventional meta-analyses using the DerSimonian-Laird random-effects model and Mantel-Haenszel fixed-effects model were performed to provide direct estimates.

## 3 Results

### 3.1 Search results

The initial search yielded 4,248 articles, and 2,628 remained after deduplication. After title/abstract screening, 1,245 irrelevant articles were removed. Full-text review was conducted for 1,383 articles, of which 1,223 were excluded for not meeting inclusion criteria and 6 were excluded due to unavailable full-text data.

Finally, 154 articles were included in the study: 153 in Chinese and 1 in English (16–169). The literature screening flow chart is shown in Figure 1.

**Figure 1:**
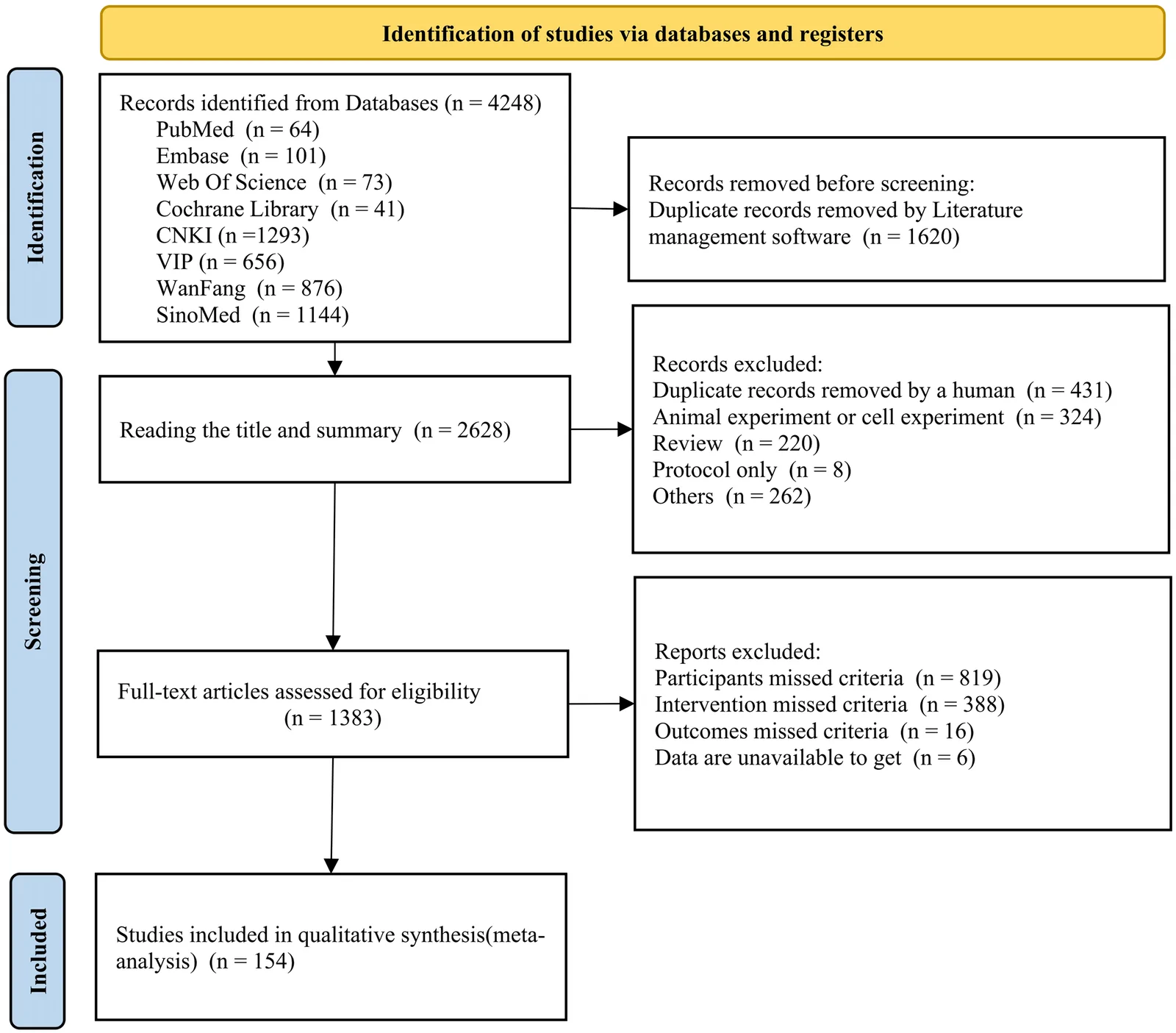
PRISMA 2020 flow diagram of study identification, screening, eligibility assessment, and inclusion.

### 3.2 Basic characteristics of included studies

A total of 154 two-arm RCTs were included, with a total sample size of 13,756 patients: 6,923 in the experimental group and 6,833 in the control group (19–172). All included RCTs provided baseline information to ensure comparability between groups. Basic characteristics of included studies are shown in Supplementary Table S3. Summary characteristics of the included randomized controlled trials by intervention in Table 1. Basic information of the six traditional Chinese medicine injections included in Supplementary Table S4.

**Table 1:** Summary characteristics of the included randomized controlled trials by intervention.

Intervention	RCTs, *n*	Participants, *n*	Duration, days	Outcome reporting, *n*
SFI + CT	42	3,178	7–90	CER 26; ADR 15; BNP 15; NT-proBNP 8; LVEF 35; LVEDD 9; 6MWT 3
SMI + CT	34	3,471	7–90	CER 24; ADR 21; BNP 11; NT-proBNP 2; LVEF 25; LVEDD 10; 6MWT 5
SHMI + CT	19	1,701	7–21	CER 15; ADR 6; BNP 2; NT-proBNP 1; LVEF 14; LVEDD 7
HQI + CT	8	751	10–28	CER 8; ADR 4; BNP 1; LVEF 7; LVEDD 1; 6MWT 1
XMLI + CT	26	2,373	7–14	CER 17; ADR 10; BNP 13; NT-proBNP 8; LVEF 20; LVEDD 5; 6MWT 5
YQFMI + CT	25	2,282	7–30	CER 15; ADR 11; BNP 1; NT-proBNP 16; LVEF 23; LVEDD 14; 6MWT 4
Total	154	13,756	7–90	CER 103; ADR 65; BNP 44; NT-proBNP 35; LVEF 124; LVEDD 45; 6MWT 17

ADR, adverse drug reaction; BNP, B-type natriuretic peptide; CER, clinical effectiveness rate; CT, conventional treatment; HQI, Huangqi injection; LVEF, left ventricular ejection fraction; LVEDD, left ventricular end-diastolic dimension; NT-proBNP, N-terminal pro-B-type natriuretic peptide; SFI, Shenfu injection; SHMI, Shengmai injection; SMI, Shenmai injection; XMLI, Xinmailong injection; YQFMI, Yiqifumai injection; 6MWT, 6-minute walk test. Outcome totals follow the manuscript narrative; intervention-specific counts were summarized from the extraction workbook.

### 3.3 Literature quality assessment

Risk-of-bias appraisal outcomes across the 154 eligible trials are laid out in Figure 2. Randomization process: 53 studies specified randomization methods (low risk); 96 studies only mentioned randomization without details (some concerns); 5 studies grouped patients by admission order (high risk) (36, 39, 48, 63, 138). Deviations from intended interventions: 5 studies mentioned double-blinding, and 1 used a placebo (low risk) (30, 43, 89, 94, 97, 126); 8 studies used single-blinding (27, 74, 86, 88, 90, 125, 141, 171), and 140 did not mention blinding (some concerns). Missing outcome data: All 154 studies had no missing outcome data (low risk). Outcome measurement: 8 studies using single blinding (which may affect measurement) were rated high risk (27, 74, 86, 88, 90, 125, 141, 171); the remaining 146 showed no measurement bias (low risk). Selective reporting: 2 studies had unreported dropouts (27, 41), 1 did not report all preset time points (98), and 1 did not report preset ADR outcomes (high risk) (39); the remaining 150 were unclear (some concerns). Other biases: 15 studies had some concerns due to high risk in other aspects (27, 36, 39, 41, 48, 63, 74, 86, 88, 90, 101, 125, 138, 141, 171); the remaining 139 showed no other biases (low risk).

**Figure 2:**
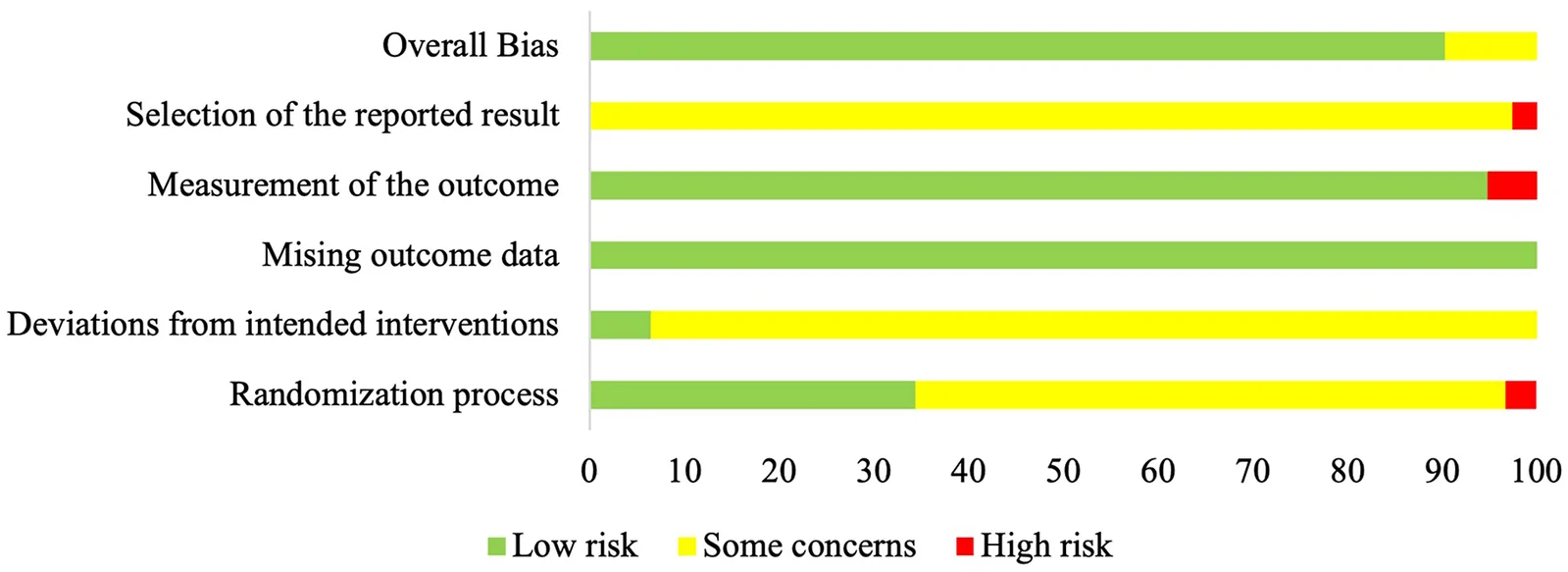
Risk of bias assessment included in the study.

### 3.4 Network analysis results

The evidence network plot for six TCMIs combined with CT in IHF treatment is shown in Figure 3. In the network diagram, each node represents a treatment regimen; the node size is proportional to the number of participants, and the thickness of the connecting lines between nodes reflects the amount of direct comparison evidence available between different treatment regimens. No closed loops formed for any of the seven outcome measures, so a consistency model was used for evaluation. Network geometry by outcomes in Supplementary Table S5. Cumulative probability heatmaps and line charts for different interventions are shown in Figures 4, 5.

**Figure 3:**
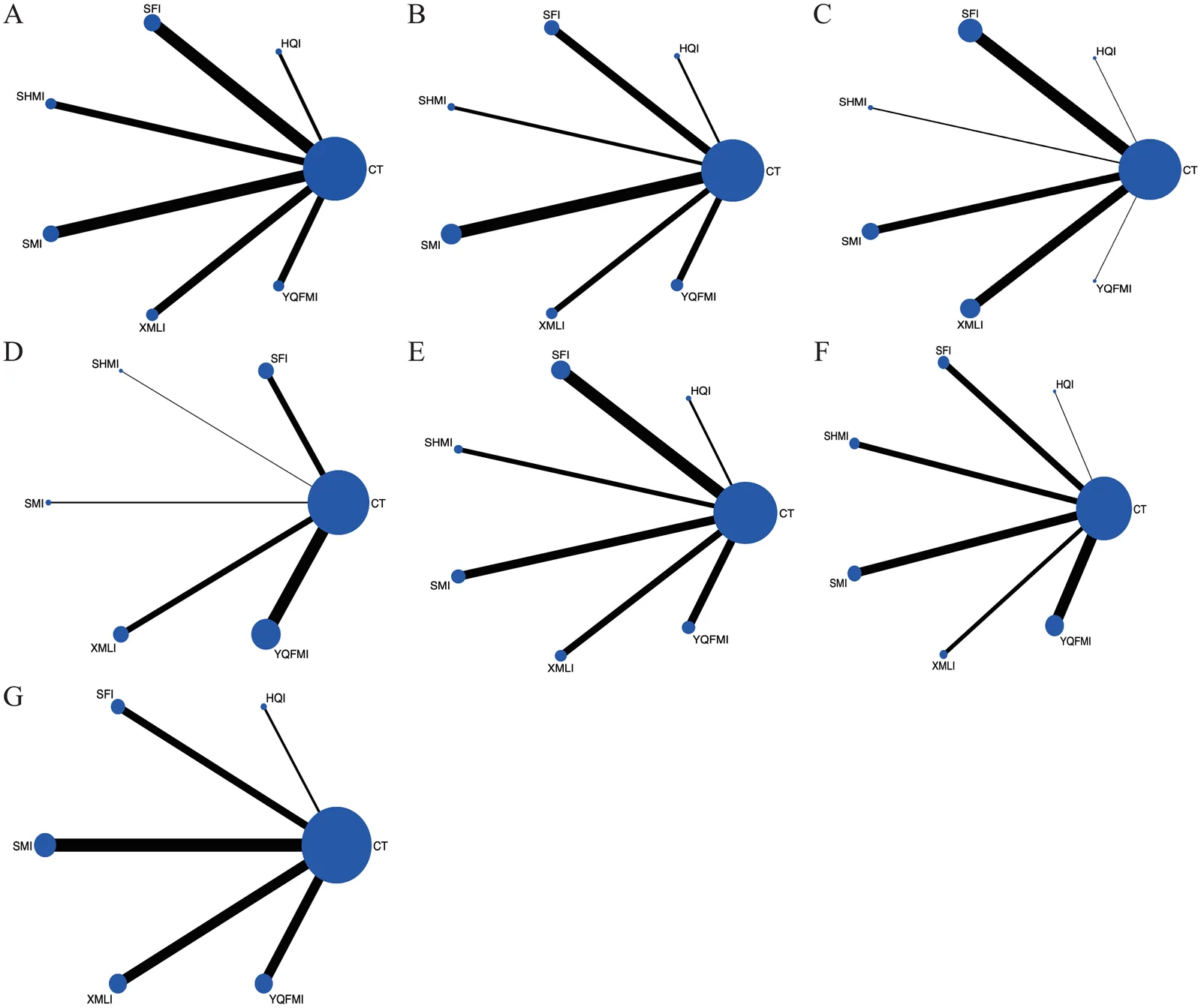
Evidence networks of each outcome. **(A)** CER, **(B)** ADRs, **(C)** BNP, **(D)** NT-pro BNP, **(E)** LVEF, **(F)** LVEDD, **(G)** 6MWT.

**Figure 4:**
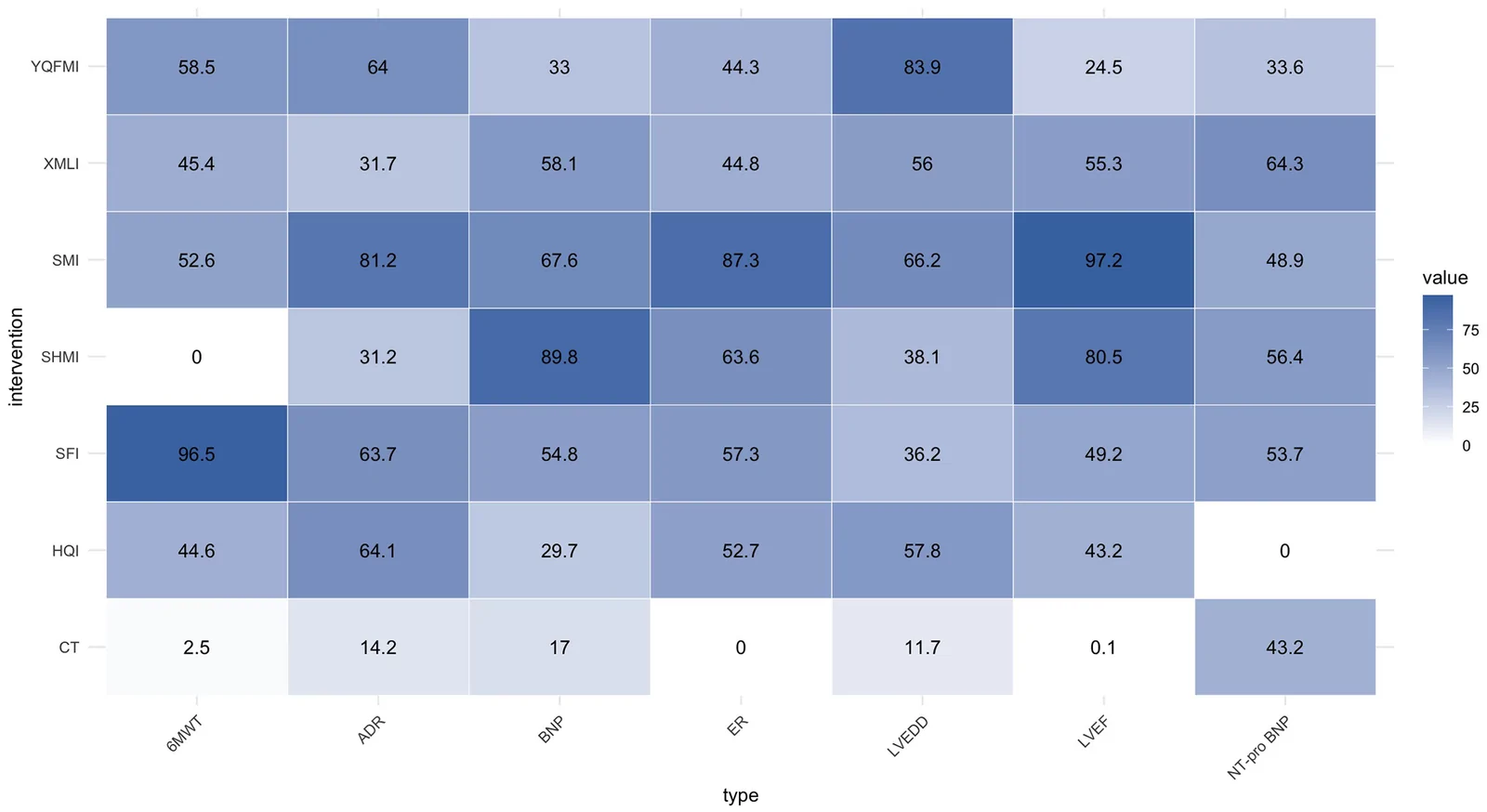
Heatmap of surface under the cumulative ranking curve (SUCRA) values across outcomes. Darker shading indicates a higher SUCRA value.

**Figure 5:**
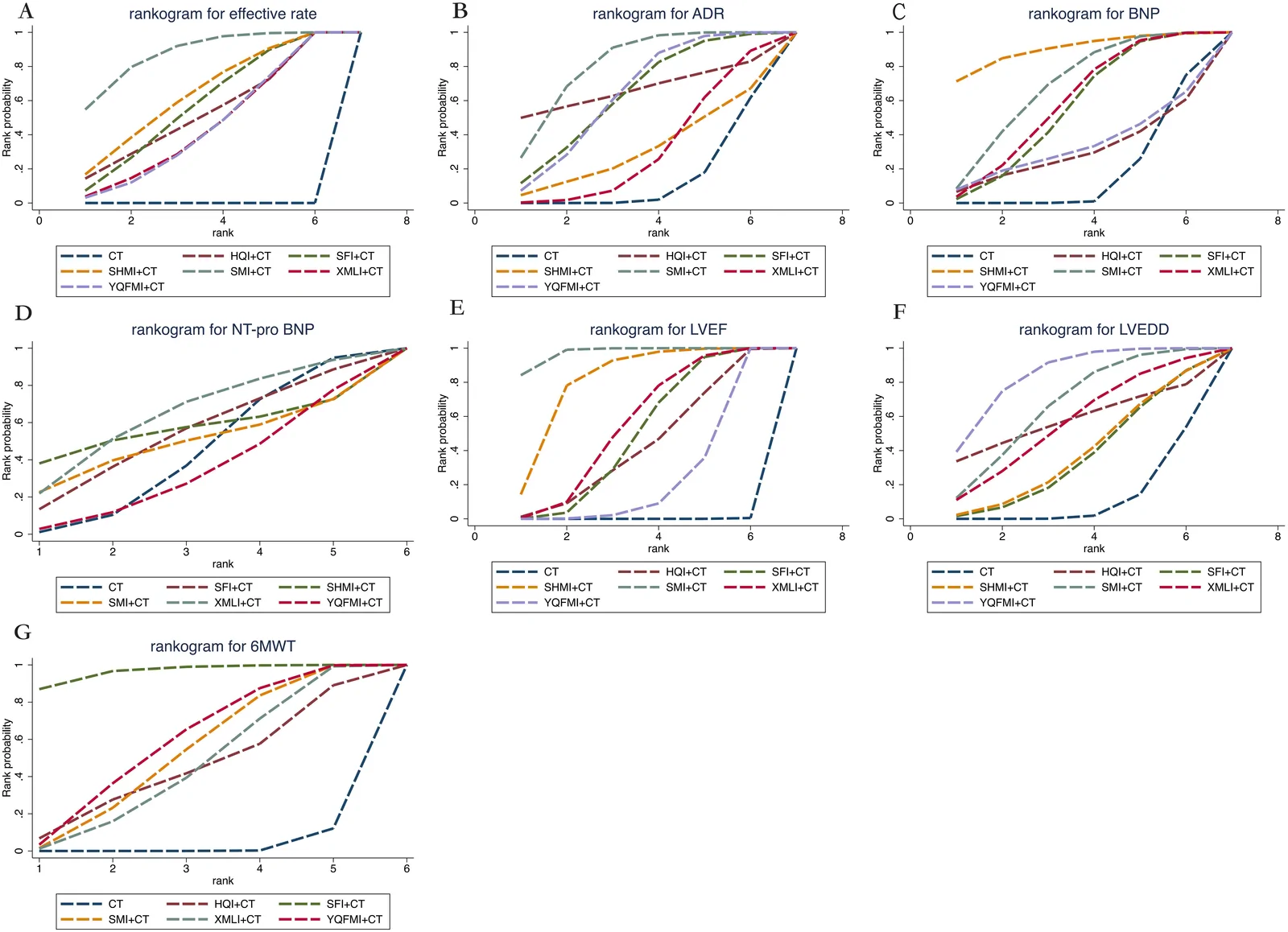
Rank probability plots for **(A)** CER, **(B)** ADRs, **(C)** BNP, **(D)** NT-pro BNP, **(E)** LVEF, **(F)** LVEDD, and **(G)** 6MWT.

#### 3.4.1 CER

A total of 103 RCTs reported CER, covering 6 TCMIs and 7 intervention regimens (20, 22, 25–27, 29, 31, 32, 35–39, 41, 44, 47–49, 52–58, 62–64, 66, 67, 69, 70, 72, 74–88, 90, 95, 96, 98–106, 110–117, 119–124, 126, 128, 129, 132, 134–136, 138–140, 143, 145–147, 151, 152, 154–159, 161, 162, 168–172). Compared with CT alone, SMI + CT [*OR*=4.29, 95% *CI* (3.33, 5.54)], SHMI + CT [*OR* = 3.91, 95% *CI* (2.79, 5.48)], SFI + CT [*OR* = 3.20, 95% CI (2.45, 4.19)], HQI + CT [OR = 3.22, 95% CI (2.05, 5.08)], XMLI + CT [OR = 4.20, 95% *CI* (3.00, 5.87)], and YQFMI + CT [*OR* = 4.73, 95% *CI* (3.23, 6.93)] all showed advantages in improving clinical symptoms/signs and cardiac function, with statistically significant differences (*P* < 0.05). SUCRA rankings placed SMI + CT (87.3%) ahead of SHMI + CT (63.6%), SFI + CT (57.3%), HQI + CT (52.7%), XMLI + CT (44.8%), and YQFMI + CT (44.3%), as shown in Figures 4, 5A, 6.

**Figure 6:**
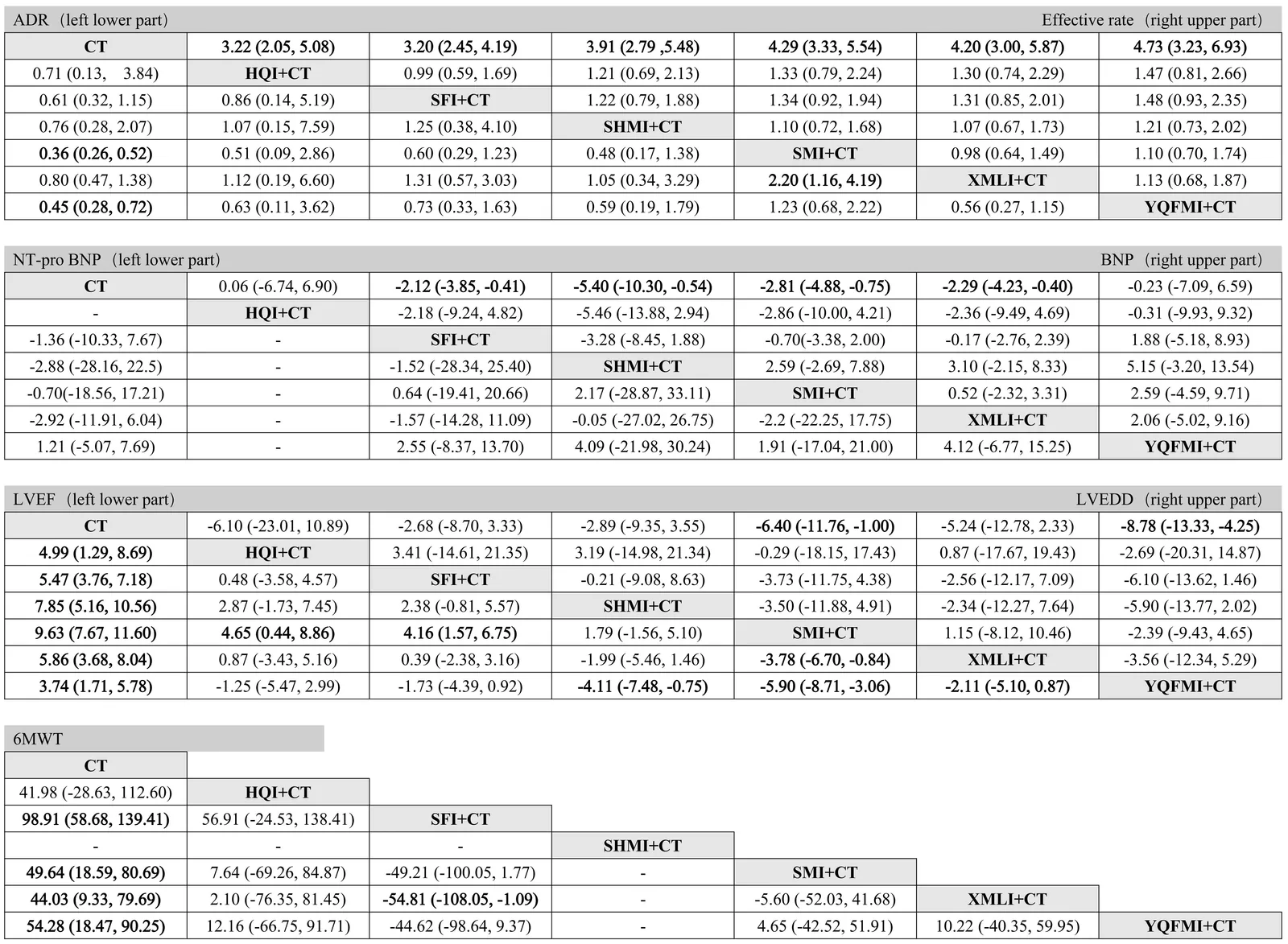
League tables for network comparisons. The upper-right triangles present CER, BBNP, and LVEDD; the lower-left triangles present ADRs, NT-pro BNP, LVEF, and 6MWT. Bold values indicate statistical significance.

#### 3.4.2 ADRs

65 RCTs reported ADR data, covering 6 TCMIs and 7 intervention regimens (21–24, 27–29, 31, 32, 34, 40, 44, 47, 52, 61–66, 69, 71, 74–79, 83, 86, 88, 92–94, 96, 99, 103, 106, 107, 110, 114, 115, 118, 119, 123, 124, 128, 129, 135, 136, 138, 140, 143, 145, 148, 153–157, 159, 161, 167, 170, 172). We have also summarized the reported adverse drug reactions separately for each TCMI to provide a clearer description of the available safety data in Table 2. 1 study reported SAE, but this study didn't descript more detail. Compared with CT alone, SMI + CT [*OR* = 0.36, 95% *CI* (0.26, 0.52)] and YQFMI + CT [*OR*=0.45, 95% *CI* (0.28, 0.72)] demonstrated benefits in reducing adverse reaction incidence, with statistically significant differences (*P* < 0.05). SMI + CT (81.2%) had the highest SUCRA value for reducing ADRs, this ranking is exploratory, as shown in Figures 4, 5B, 6.

**Table 2:** Distribution of adverse event types by intervention.

Treatment	Headeche Or Dizziness(n)	Arrhythmia Or Palpitation(n)	Nausea or vomiting(n)	Hypotension(n)	Gastrointestinal reaction(n)	Hypersensitivity(n)	Hepatic or renal dysfunction(n)	Arthralgia(n)	Fatigue(n)	Worsening Heart Failure(n)	PCI or CABG	Hyperkalemia(n)	AE-unspecified(n)	SAE
SFI	1	4	2	—	—	2	5	—	—	1	—	—	—	—
SMI	18	5	2	3	—	4	—	2	—	5	7	—	4	1
SHMI	2	—	1	—	1	2	—	—	—	—	—	—	—	—
HQI	—	—	—	1	—	—	—	—	—	—	—	—	—	—
XMLI	12	1	7	—	—	7	—	—	7	—	—	—	1	—
YMFMI	2	3	13	1	3	1	1	—	—	—	—	1	1	—

#### 3.4.3 BNP

44 RCTs reported BNP levels, covering 6 TCMIs and 7 intervention regimens (23, 27–29, 31, 33, 36, 37, 46, 50, 51, 55–58, 60, 61, 69, 77, 80–83, 86, 88, 89, 91, 104, 108, 114, 122, 123, 127, 129, 130, 132, 134, 135, 142, 144, 145, 169). Compared with CT alone, SHMI + CT [*SMD* = −5.40, 95% *CI* (−10.30, −0.54)], SMI + CT [*SMD* = −2.81, 95% *CI* (−4.88, −0.75)], XMLI + CT [*SMD* = −2.29, 95% *CI* (−4.23, −0.40)], and SFI + CT [*SMD* = −2.12, 95% *CI* (−3.85, −0.41)] delivered further BNP reductions, with statistically significant differences (*P* < 0.05). SUCRA results showed SHMI + CT (89.8%), SMI + CT (67.6%), XMLI + CT (58.1%), and SFI + CT (54.8%) had the higher rank, as shown in Figures 4, 5C, 6. However, the estimate was based on indirect evidence and should be interpreted cautiously.

#### 3.4.4 NT-proBNP

35 RCTs reported NT-proBNP levels, covering 5 TCMIs and 6 intervention regimens (19, 21, 34, 35, 39, 40, 42, 44, 64, 73, 109, 126, 128, 133, 137, 138, 141, 143, 147–149, 152, 153, 156, 158, 160–163, 165–167, 170–172). None of the TCMI add-on regimens reached statistical significance in reducing NT-proBNP compared with CT alone. XMLI + CT(64.3%) ranked highest according to SUCRA; however, the estimate was based on indirect evidence and should be interpreted cautiously, as shown in Figures 4, 5D, 6.

#### 3.4.5 LVEF

A total of 124 RCTs reported LVEF data, covering 6 TCMIs and 7 intervention regimens (20–28, 30–33, 36–38, 40–43, 45–48, 50–66, 68, 69, 73, 77–81, 83–89, 91–97, 99–102, 104–110, 114–118, 120, 121, 123–128, 130–133, 135–137, 139–141, 143–145, 147–151, 153–166, 168–172). Compared with CT alone, SMI + CT [*MD* = 9.63, 95% *CI* (7.67, 11.60)], SHMI + CT [*MD* = 7.85, 95% *CI* (5.16, 10.56)], XMLI + CT [*MD* = 5.86, 95% *CI* (3.68, 8.04)], SFI + CT [*MD* = 5.47, 95% *CI* (3.76, 7.18)], HQI + CT [*MD* = 4.99, 95% *CI* (1.29, 8.69)], and YQFMI + CT [*MD* = 3.74, 95% *CI* (1.71, 5.78)] yielded significant LVEF improvements, with statistically significant differences (*P* < 0.05). SUCRA values showed SMI + CT (97.2%) had the highest rank for improving LVEF, followed by SHMI + CT (80.5%), XMLI + CT (55.3%), SFI + CT (49.2%), HQI + CT (43.2%), and YQFMI + CT (24.5%), as shown in Figures 4, 5E, 6.

#### 3.4.6 LVEDD

45 RCTs reported LVEDD, covering 6 TCMIs and 7 intervention regimens (22, 27, 28, 31, 38, 43, 50, 60, 62, 64, 69, 77, 80, 81, 83, 86, 87, 92, 100–102, 104–106, 109, 115, 123, 128, 133, 137, 145, 148–151, 153–157, 162, 164–166, 169). Compared with CT alone, YQFMI + CT [*MD* = −8.78, 95% *CI* (−13.33, −4.25)] and SMI + CT [*MD* = −6.40, 95% *CI* (−11.76, −1.00)] were associated with greater LVEDD reductions, with statistically significant differences (*P* < 0.05). Based on the SUCRA analysis, combination therapy with YQFMI + CT was the most effective intervention for reducing LVEDD in patients, with a SUCRA value of 83.9%, followed by SMI + CT (66.2%), as shown in Figures 4, 5F, 6.

#### 3.4.7 6MWT

17 RCTs reported 6MWT data, covering 5 TCMIs and 6 intervention regimens (30, 36, 50, 79, 86, 87, 89, 94, 114, 126–128, 145, 149, 162, 165, 166). Compared with CT alone, SFI + CT [*MD* = 98.91, 95% *CI* (58.68, 139.41)], YQFMI + CT [*MD* = 54.28, 95% *CI* (18.47, 90.25)], SMI + CT [*MD* = 49.64, 95% *CI* (18.59, 80.69)], and XMLI + CT [*MD* = 44.03, 95% *CI* (9.33, 79.69)] significantly improved 6MWT performance, with statistically significant differences (*P* < 0.05). Based on the SUCRA analysis, combination therapy with SFI + CT(66.2%) was the most effective intervention for improving 6WMT in patients, followed by YQFMI + CT (58.5%), SMI + CT (52.6%), and XMLI + CT (45.4%), as shown in Figures 4, 5G, 6.

### 3.5 Sensitivity analysis

Sensitivity analyses using lower and higher correlation coefficients produced broadly similar effect estimates and did not materially change the direction of the main findings. Treatment rankings were also generally stable, indicating that the main conclusions were not materially driven by the assumed within-study correlations. Sensitivity analyses using alternative pre-post correlation coefficients in Supplementary Table S6.

The other sensitivity analysis enrolled 49 RCTs with ≥100 patients per study (total *n* = 6,298). Results showed that: CER and LVEDD findings remained consistent with the primary analysis; For ADRs, discrepancies were observed between original and sensitivity estimates for SHMI + CT vs. CT and SMI + CT vs. CT; For LVEF, differences between original and sensitivity results were found in the following pairwise comparisons: HQI + CT, SFI + CT, YQFMI + CT vs. CT; SMI + CT vs. HQI + CT, SFI + CT; XMLI + CT vs. SMI + CT; YQFMI + CT vs. SHMI + CT, XMLI + CT; For 6MWT, discrepancies were seen for SFI + CT, XMLI + CT, YQFMI + CT vs. CT, and XMLI + CT vs. SFI + CT. In the original NMA, SMI + CT was the top-ranked regimen for LVEF improvement, consistent with sensitivity analysis findings. The best regimens for improving clinical symptoms, reducing ADRs, lowering BNP, and improving 6MWT were SMI + CT, SMI + CT, SHMI + CT, and SFI + CT respectively in the primary analysis, vs. SHMI + CT, YQFMI + CT, SMI + CT, and SMI + CT in the sensitivity analysis. As seen in Supplementary Figure S1.

### 3.6 Publication bias

All seven outcomes were supported by more than 15 RCTs, so funnel plots (Figure 7**)** were generated to assess publication bias across endpoints. Dots in distinct colors represent pairwise comparisons between different intervention regimens. Results showed study data were roughly asymmetrically distributed on either side of the vertical line at x = 0, with a substantial angle between the fitted curve and the vertical reference line—suggesting potential publication bias. This bias may stem from underreporting of negative results and a scarcity of large-scale RCTs.

**Figure 7:**
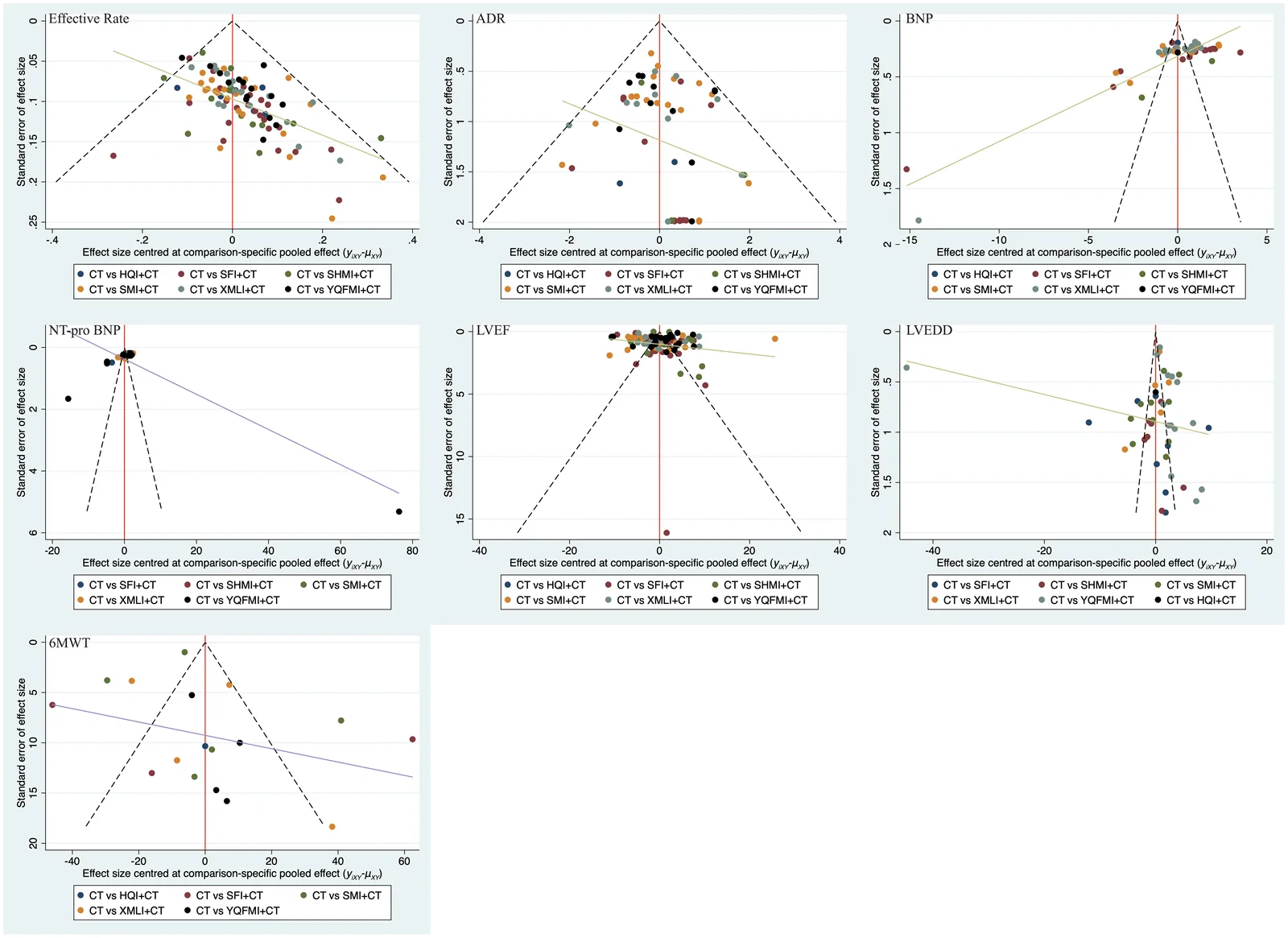
Comparison-adjusted funnel plots for the seven outcomes. Asymmetry suggests possible small-study effects or publication bias.

### 3.7 Evidence quality evaluation

CINeMA generated 40 direct comparisons and 95 indirect comparisons in total. Of these, 41 were rated as “low” quality of evidence, and 94 as “very low”. Owing to the lack of negative results and large-scale RCTs, “reporting bias” was rated as “some concerns” for most studies. For certain outcomes, the limited number of comparable trials raised “major concerns” regarding imprecision. The absence of closed loops in the evidence network led to insufficient transitivity, giving rise to “major concerns” around incoherence. Overall evidence quality was downgraded due to the above factors, resulting in final ratings of “low” or “very low”. CINeMA results in Supplementary Table S7.

### 3.8 Overall meta-analysis and heterogeneity assessment

Conventional pairwise meta-analyses were conducted for all outcome measures across different interventions. Results showed low heterogeneity for CER (*I*^2^ = 0%) and ADRs (*I*^2^ = 9.30%); high heterogeneity was observed for BNP (*I*^2^ = 97.00%), NT-proBNP (*I*^2^ = 97.60%), LVEF (*I*^2^ = 99.70%), LVEDD (*I*^2^ = 99.80%), and 6MWT (*I*^2^ = 94.40%).

Subgroup analyses were performed for high-heterogeneity outcomes based on mean age, intervention duration, treatment dosage, baseline ejection fraction, and IHF classification. Subgroup findings indicated: BNP reduction benefits were more pronounced in the 14-day intervention, mean age 65 years, high-dose subgroups, and chronic IHF; NT-pro BNP showed larger benefits in the 14-day intervention, mean age 65 years, high-dose subgroups, and acute IHF; LVEF improvements were greater in the 14-day intervention, mean age 65 years, low-dose, baseline LVEF 40%, and acute IHF subgroups; LVEDD reductions were more substantial in the 14-day intervention, mean age 65 years, high-dose subgroups, and chronic IHF; 6MWT performance gains were better in the 14-day intervention, mean age 65 years, and low-dose subgroups. No definitive source of heterogeneity was identified through subgroup analysis. Leave-one-out sensitivity analysis did not reveal the source of heterogeneity. Several factors may contribute to it. BNP and NT-proBNP levels may be influenced by assay platforms, detection methods, and patient renal function; LVEF and LVEDD measurements could be affected by ultrasound equipment, timing of measurement, and operator experience; as a functional endpoint, 6MWT may additionally be shaped by patient exercise tolerance, rehabilitation status, comorbidities, and variations in testing protocols. Overall and subgroup meta-analysis results in Supplementary Table S8.

## 4 Discussion

The present study pooled data from 154 RCTs involving 13,756 IHF patients. All enrolled patients received either CT alone or CT combined with one of the six TCMIs. By directly comparing TCMI + CT vs. CT alone, and indirectly comparing the efficacy and safety of the six TCMIs (SFI, SMI, SHMI, HQI, XMLI, YQFMI), this study provides reference for selecting appropriate TCMIs as adjuvant therapy for IHF patients. This results suggested that: (1) Compared with CT alone, add-on TCMIs further improved clinical symptoms and cardiac function, reduced BNP levels, improved exercise tolerance in IHF patients, but yielded uncertain effects on NT-proBNP, without increasing adverse events in short-time safety. (2) SUCRA rankings showed that SMI + CT had the highest ranking probability in improving clinical symptoms and LVEF; SHMI + CT and XMLI + CT ranked highest in reducing BNP and NT-proBNP levels, respectively; YQFMI + CT ranked highest for reducing LVEDD; and SFI + CT delivered the highest rank notable improvement in 6MWT. The estimate was based on indirect evidence and should be interpreted cautiously. The differential efficacy across TCMIs reflects the unique strengths of their multi-component, multi-target mechanisms in comprehensive IHF treatment. (3) CINeMA evidence quality assessment indicated that the overall quality of included literature remains to be improved, and clinicians are advised to carefully select combination regimens based on individual patient conditions. While several previous NMAs have explored TCMI + CT regimens for HF, the present study focuses specifically on IHF of ischemic etiology rather than the broader HF population, and applies strict inclusion/exclusion criteria to ensure consistency in the enrolled patient population. We also standardized the definition of clinical effectiveness rate across outcomes to ensure consistency of pooled results. To further enhance the reliability of our findings, we conducted sensitivity and subgroup analyses to explore the impact of relevant variables on efficacy, and assessed study quality using the RoB 2.0 and CINeMA framework.

As the end-stage of progressive IHD, IHF onset and progression involve multiple pathological mechanisms, including myocardial ischemia-reperfusion injury, inflammatory responses, oxidative stress, and abnormal energy metabolism. These pathways interact and amplify one another, creating a vicious cycle that progressively worsens cardiac function. Compared with HF caused by non-ischemic etiologies, IHF is associated with higher all-cause mortality and poorer prognosis (173). Although medications such as ARNIs and SGLT2is have been widely used in clinical HF management and improved patient prognosis in recent years, some patients cannot tolerate guideline-recommended regimens due to hypotension, renal insufficiency, or other factors, leaving their clinical treatment needs unmet (174). TCMIs are traditional Chinese medicine preparations developed by combining TCM theory with modern pharmaceutical technology. Featuring multi-component, multi-target effects, rapid onset, and high bioavailability, they have been shown in multiple clinical studies to improve clinical symptoms, exercise tolerance, and quality of life in HF patients (175). By combining Bayesian NMA with SUCRA ranking, this study provides a reference for selecting TCMI regimens in clinical IHF management.

Compared with CT, add-on SMI further improved clinical symptoms and LVEF levels. SMI is a compound preparation of Hong Shen (red ginseng) and Mai Dong (Ophiopogon japonicus), with the TCM efficacy of replenishing qi and nourishing yin. Chronic ischemia disrupts oxygen supply-demand balance in cardiomyocytes of IHF patients. ATP depletion triggers intracellular calcium overload and oxygen free radical damage; imbalanced free radical metabolism causes mitochondrial oxidative damage, impairing normal energy conversion in cardiomyocytes. Meanwhile, persistent ischemia induces chronic immune-inflammatory responses and endothelial homeostasis dysregulation. These interconnected processes collectively exacerbate myocardial dysfunction (176). Previous studies have shown that SMI preconditioning reduces the expression of nuclear transcription factor NF-*κ*B p65 in myocardial nuclei of rats with ischemia-reperfusion injury, decreases phosphorylation of inhibitor of nuclear factor kappa-B (I*κ*B), and elevates I*κ*B levels. It also exerts anti-inflammatory, antioxidant, and anti-apoptotic effects by regulating the protein expression of NADPH oxidase isoform gp91phox, mitochondrial superoxide dismutase 2 (SOD2), catalase (CAT), and apoptosis-related markers phosphorylated c-Jun N-terminal kinase (p-JNK) and Caspase-3, thereby protecting the myocardium in IHF (176). In addition, SMI maintains mitochondrial functional stability and normal energy metabolism in cardiomyocytes by increasing pyruvate dehydrogenase (PDH) activity and reducing pyruvate accumulation. LVEF is a key indicator of cardiac systolic function; in IHF patients, cardiomyocyte necrosis reduces the number of functional contractile myocytes, lowers cardiac contractility, and decreases LVEF (177). LVEF improvement is often correlated with symptom relief. SMI alleviates clinical symptoms in IHF patients through anti-inflammatory, antioxidant, anti-apoptotic, and myocardial energy metabolism-regulating effects, while also promoting the recovery of left ventricular systolic function. SMI also increases cAMP concentrations by reducing Ca^2+^ reuptake into myocardial mitochondria, which accelerates myocardial relaxation rate and shortens ventricular diastolic interval, thereby better ensuring ventricular filling in patients.

BNP and NT-proBNP are proteolytic products of precursor proteins produced by cardiomyocytes under stress, pressure, or volume overload. They are among the most important biomarkers for assessing IHF pathological status, and elevated levels reflect HF severity and prognostic outcomes. Multiple guidelines recommend BNP and NT-proBNP as diagnostic and exclusion criteria for HF, and NT-proBNP level change rate is used as a primary surrogate endpoint in HF-related clinical studies. A systematic review of 19 studies found that for every 100 ng/L increase in admission BNP level, the relative risk of all-cause mortality in HF patients rises by 35% (178). A study of 599 patients reported that the mortality rate of patients with NT-proBNP > 986 pg/mL was 2.88 times higher than that of patients with levels ≤986 pg/mL (179). Our findings suggest that add-on SHMI is a superior option for further reducing BNP levels compared with CT alone, and holds promise for improving prognosis and slowing disease progression in IHF patients. SHMI is derived from the classic TCM formula Shengmai San, composed of red ginseng, Ophiopogon japonicus, and Schisandra chinensis, with the effects of replenishing qi and nourishing yin, restoring pulse, and relieving desertion syndrome. Basic research indicated that it activates the AMPK signaling pathway via an energy-dependent mechanism, protects mitochondrial function, increases levels of ATP, ADP, and phosphocreatine, and upregulates the expression of factors related to free fatty acid oxidation, glucose oxidation, and mitochondrial biogenesis, thereby inhibiting angiotensin II (Ang II)-induced cardiomyocyte hypertrophy and apoptosis (180). Ang II is a key component of the renin-angiotensin-aldosterone system (RAAS). It regulates aldosterone secretion, increases water and sodium reabsorption, raises systemic blood volume, and increases cardiac volume load, further exacerbating HF. By means of inhibiting Ang II-induced cardiomyocyte apoptosis, SHMI reduces cardiac volume load and lowers BNP levels. Real-world clinical application analyses of SHMI suggest that its combination with diuretics and vasodilators can further improve cardiac function and clinical symptoms in HF patients with qi-yin deficiency and blood stasis syndrome (181, 182). For NT-proBNP, none of the TCMI add-on regimens reached the preset statistical significance threshold compared with CT alone, Where these intervals were wide, they indicated uncertainty about the magnitude and potentially the direction of the treatment effect. Combined with SUCRA results indicates that among the six TCMIs, XMLI + CT ranked highest for reducing NT-proBNP levels. XMLI is a compound peptide injection extracted from Periplaneta americana, mainly containing compound amino acids, nucleosides, and polypeptides. Combined transcriptomic and proteomic analyses suggest that it exerts cardioprotective effects by activating the cGMP-PKG signaling pathway, downregulating profibrotic genes such as COL1A1 and MMP-9, and modulating key proteins including sGC, PKG, and PDE5A (183). Another systematic review of XMLI for chronic heart failure (CHF) found that add-on XMLI further reduced NT-proBNP levels in CHF patients compared with CT. The lack of statistical significance in our study may be attributed to insufficient sample size of included literature, which leads to low statistical power, wide confidence intervals, and reduced test efficiency. More large-scale clinical studies are needed in the future to further evaluate the effect of XMLI + CT on NT-proBNP reduction.

Ventricular remodeling is a key pathological process in IHF that accelerates IHF progression and further worsens cardiac function. It involves multiple pathological mechanisms, including cardiomyocyte necrosis, apoptosis, and myocardial fibrosis. LVEDD is a critical indicator for assessing left ventricular structure. Our findings indicate that YQFMI + CT has the highest ranking probability for reducing LVEDD. YQFMI is composed of red ginseng, Ophiopogon japonicus, and Schisandra chinensis. It shares the same components as SMI but differs in preparation form, and also has the effects of replenishing qi and nourishing yin, restoring pulse, and relieving desertion syndrome. Multiple omics studies have revealed the mechanisms by which YQFMI inhibits ventricular remodeling. In terms of proteomics, it enhances mitochondrial function in cardiomyocytes, improves oxidative phosphorylation and myocardial energy metabolism, and reduces cardiomyocyte apoptosis by regulating PGC-1*α* and its downstream effectors (184). Metabolomic results show that it regulates pathways including valine, leucine, and isoleucine biosynthesis, taurine and hypotaurine metabolism, histidine metabolism, and arginine biosynthesis. It upregulates 55 differential metabolites such as uric acid and creatine, thereby inhibiting myocardial fibrosis and ventricular remodeling (185).

The 6MWT reflects a patient's exercise tolerance and functional status, and is an important indicator for evaluating quality of life in HF patients. Our results show that SFI + CT has the highest SUCRA value in improving 6MWT performance. SFI is composed of red ginseng and aconite slices, with the TCM effects of restoring yang to rescue desertion and replenishing qi to consolidate collapse. Reduced exercise tolerance in IHF patients is closely associated with functional abnormalities such as decreased cardiac output (CO), impaired energy metabolism, and insufficient tissue perfusion. Basic research reveals that SFI reduces myocardial inflammatory responses by regulating signaling pathways such as NF-*κ*B and HMGB1/TLR4/NF-*κ*B, and improves myocardial energy metabolism by regulating calcium homeostasis and repairing mitochondrial damage, thereby exerting cardioprotective effects and improving cardiac function (186–188). As cardiac function improves, CO increases and tissue perfusion is enhanced, which helps boost exercise tolerance in IHF patients.

In terms of safety, the included studies strictly limited TCMI dosage and treatment duration. Overall, no increase in the incidence of serious drug-related adverse events was observed in short-time. We summarized the reported adverse drug reactions separately for each TCMI to provide a clearer description of the available safety data in Table 2. A wider variety of ADRs was reported, including headache or dizziness, arrhythmia or palpitation, nausea or vomiting, hypotension, gastrointestinal reaction, hypersensitivity, hepatic or renal dysfunction, arthralgia, fatigue, cough, worsening HF, PCI or CABG, hyperkalemia. Current evidence is not sufficient to comprehensively evaluate the long-term safety of TCMIs + CT for IHF. It should be noted that adverse event reporting in relevant studies is insufficient, with major deficiencies in event definitions, collection procedures, timing, severity grading, and attribution. Most studies focused on short-term safety only, with inadequate renal function monitoring, particularly concerning for cardiorenal assessment in IHF. Given the special administration route of TCMIs and previous reports of allergic reactions and infusion-related adverse events, future studies should document adverse event occurrences in detail and attach importance to relevant data, to provide reliable evidence for clinicians using TCMIs in practice. Meanwhile, in clinical TCMI application, dosage, infusion rate, and treatment duration should be strictly controlled per the drug instructions. Drug selection should be based on patient symptoms, and TCMIs should be reasonably combined with Western medications to avoid potential drug interactions (189). Higher-quality studies are still needed in the future to continuously monitor and evaluate the long-term safety of TCMI + CT regimens.

## 5 Limitations

This study has the following limitations: (1) The methodological quality of included studies was generally low. Most studies did not report details on random sequence generation, allocation concealment, or blinding, which may introduce selection bias and performance bias. All SUCRA-based rankings are presented as exploratory and hypothesis-generating and are interpreted alongside effect estimates; (2) Outcome selected was clinical effectiveness rate, a soft endpoint, with no evaluation of hard endpoints. Also, heterogeneity in outcome measures affected the reliability of study findings to some extent; (3) Most included studies did not match intravenous fluid volumes between the TCMI intervention and control groups. Careful control of fluid loading is a central consideration in the management of heart failure, particularly acute decompensated IHF. All evaluated TCMIs were administered intravenously, introducing additional intravenous fluid into the intervention groups, whereas the control groups received conventional treatment alone without the corresponding infusion volume. This difference in fluid loading could influence outcomes such as cardiac function and NT-proBNP levels and potentially bias the estimated treatment effects. (4)Most studies did not report patient prognosis data (including hospitalization rate and cardiovascular event rate) or long-term follow-up data, which limits the evaluation of TCMI effects on prognosis and long-term safety; (5) All included studies were conducted in mainland China and involved only a single ethnic group, resulting in low generalizability of the findings; (6) Currently, only indirect comparisons exist between the six TCMIs, with wide confidence intervals, which may lead to false-negative results; (7) Most included studies did not report syndrome differentiation of subjects, which somewhat affects the reliability of the conclusions; (8)Because within-study baseline-to-follow-up correlations were rarely reported, change-score variances required imputation in some trials. Although sensitivity analyses across plausible correlation values were undertaken, residual uncertainty related to imputation remains and should be considered when interpreting continuous-outcome estimates and rankings.

Future studies are expected to focus on the following aspects to further explore the efficacy and safety of TCMI + CT for IHF: First, high-quality clinical trials with randomized, triple-blind, placebo-controlled designs are needed. Meanwhile, future RCTs of TCMIs for IHF should match total intravenous fluid between groups to embalance this bias; Second, clinical trial reports should follow standardized protocols, and baseline characteristics (including specific drugs and dosages used in CT) should be documented in detail; Finally, in addition to clinical outcomes such as clinical effectiveness rate, HF biomarkers, and cardiac function indicators, prognostic measures including rehospitalization rate, cardiovascular mortality, and all-cause mortality should also be evaluated.

## 6 Conclusion

This study suggests that TCMI combined with CT can further inhibit ventricular remodeling in IHF patients, improve their clinical symptoms, exercise tolerance, and quality of life, with favorable short-term safety. It gives an adjuvant treatment option for clinical IHF management. Yet, given the generally poor methodological quality of included studies, lack of direct head-to-head comparisons, and insufficient safety data reporting, no definitive conclusions can be drawn regarding the absolute ranking of drugs or their long-term safety at present. More rigorous, standardized, high-quality clinical RCTs and long-term follow-up studies are still needed to strengthen the evidence base for evaluating the efficacy and safety of TCMIs in IHF intervention.

## Data Availability

The datasets presented in this study can be found in online repositories. The names of the repository/repositories and accession number(s) can be found in the article/Supplementary Material.
