# Supplementary material for: Comparative efficacy and safety of six traditional Chinese medicine injections for ischemic heart failure: a systematic review and Bayesian network meta-analysis
Source: Front Cardiovasc Med. 2026 Oct 30;13:1969858. doi: 10.3389/fcvm.2026.1969858 (PMC13759412; doi:10.3389/fcvm.2026.1969858)
Supplement: Supplementary file 1 [file Supplementaryfile1.docx]

Supplementary Materials

This file contains six supplementary tables and one supplementary figure.

# 1 Supplementary Tables

Supplementary Table S1. PRISMA network meta-analysis checklist.

Supplementary Table S2. Search strategies for the network meta-analysis.

Supplementary Table S3. Basic characteristics of the 154 randomized controlled trials included in the network meta-analysis.

Supplementary Table S4. Basic information on the six traditional Chinese medicine injections included in the analysis.

Supplementary Table S5. Network geometry by outcomes.

Supplementary Table S6. Sensitivity analyses using alternative pre–post correlation coefficients.

Supplementary Table S7. Confidence ratings from CINeMA assessments.

Supplementary Table S8. Overall and subgroup meta-analysis results.

# 2 Supplementary Figure

Supplementary Figure S1. League tables from the sensitivity analyses.

# 1 Supplementary Tables

**Supplementary Table S1.** PRISMA network meta-analysis checklist.

| **Section/Topic** | **Item #** | **Checklist Item** | **Reported on Page #** |
| --- | --- | --- | --- |
| **TITLE** |  |  |  |
| Title | 1 | Identify the report as a systematic review incorporating a network meta-analysis (or related form of meta-analysis). | 1 |
|  |  |  |  |
| **ABSTRACT** |  |  |  |
| Structured summary | 2 | Provide a structured summary including, as applicable:  Background: main objectives  Methods: data sources; study eligibility criteria, participants, and interventions; study appraisal; and synthesis methods, such as network meta-analysis.  Results: number of studies and participants identified; summary estimates with corresponding confidence/confidence intervals; treatment rankings may also be discussed. Authors may choose to summarize pairwise comparisons against a chosen treatment included in their analyses for brevity.  Discussion/Conclusions: limitations; conclusions and implications of findings.  Other: primary source of funding; systematic review registration number with registry name. | 1 |
|  |  |  |  |
| **INTRODUCTION** |  |  |  |
| Rationale | 3 | Describe the rationale for the review in the context of what is already known, including mention of why a network meta-analysis has been conducted. | 2 |
| Objectives | 4 | Provide an explicit statement of questions being addressed, with reference to participants, interventions, comparisons, outcomes, and study design (PICOS). | 3-4 |
|  |  |  |  |
| **METHODS** |  |  |  |
| Protocol and registration | 5 | Indicate whether a review protocol exists and if and where it can be accessed (e.g., Web address); and, if available, provide registration information, including registration number. | 2 PROSPERO：CRD420261380716 |
| Eligibility criteria | 6 | Specify study characteristics (e.g., PICOS, length of follow-up) and report characteristics (e.g., years considered, language, publication status) used as criteria for eligibility, giving rationale. Clearly describe eligible treatments included in the treatment network, and note whether any have been clustered or merged into the same node (with justification). | 2-3 |
| Information sources | 7 | Describe all information sources (e.g., databases with dates of coverage, contact with study authors to identify additional studies) in the search and date last searched. | 2-3 |
| Search | 8 | Present full electronic search strategy for at least one database, including any limits used, such that it could be repeated. | Supplementary Table 2‌ |
| Study selection | 9 | State the process for selecting studies (i.e., screening, eligibility, included in systematic review, and, if applicable, included in the meta-analysis). | 2-3 |
| Data collection process | 10 | Describe method of data extraction from reports (e.g., piloted forms, independently, in duplicate) and any processes for obtaining and confirming data from investigators. | 2-3 |
| Data items | 11 | List and define all variables for which data were sought (e.g., PICOS, funding sources) and any assumptions and simplifications made. | 2-3 |
| Geometry of the network | S1 | Describe methods used to explore the geometry of the treatment network under study and potential biases related to it. This should include how the evidence base has been graphically summarized for presentation, and what characteristics were compiled and used to describe the evidence base to readers. | 3 |
| Risk of bias within individual studies | 12 | Describe methods used for assessing risk of bias of individual studies (including specification of whether this was done at the study or outcome level), and how this information is to be used in any data synthesis. | 3 |
| Summary measures | 13 | State the principal summary measures (e.g., risk ratio, difference in means). Also describe the use of additional summary measures assessed, such as treatment rankings and surface under the cumulative ranking curve (SUCRA) values, as well as modified approaches used to present summary findings from meta-analyses. | 3 |
| Planned methods of analysis | 14 | Describe the methods of handling data and combining results of studies for each network meta-analysis. This should include, but not be limited to:  Handling of multi-arm trials;  Selection of variance structure;  Selection of prior distributions in Bayesian analyses; and  Assessment of model fit. | 3 |
| Assessment of Inconsistency | S2 | Describe the statistical methods used to evaluate the agreement of direct and indirect evidence in the treatment network(s) studied. Describe efforts taken to address its presence when found. | 3 |
| Risk of bias across studies | 15 | Specify any assessment of risk of bias that may affect the cumulative evidence (e.g., publication bias, selective reporting within studies). | 3 |
| Additional analyses | 16 | Describe methods of additional analyses if done, indicating which were pre-specified. This may include, but not be limited to, the following:  Sensitivity or subgroup analyses;  Meta-regression analyses;  Alternative formulations of the treatment network; and  Use of alternative prior distributions for Bayesian analyses (if applicable). | 3 |
| **RESULTS†** |  |  |  |
| Study selection | 17 | Give numbers of studies screened, assessed for eligibility, and included in the review, with reasons for exclusions at each stage, ideally with a flow diagram. | 3-4；Figure1 |
| Presentation of network structure | S3 | Provide a network graph of the included studies to enable visualization of the geometry of the treatment network. | Figure 3 |
| Summary of network geometry | S4 | Provide a brief overview of characteristics of the treatment network. This may include commentary on the abundance of trials and randomized patients for the different interventions and pairwise comparisons in the network, gaps of evidence in the treatment network, and potential biases reflected by the network structure. | 4,Supplementary Table 5 |
| Study characteristics | 18 | For each study, present characteristics for which data were extracted (e.g., study size, PICOS, follow-up period) and provide the citations. | Supplementary Table 3 |
| Risk of bias within studies | 19 | Present data on risk of bias of each study and, if available, any outcome level assessment. | 9,Figure 2 |
| Results of individual studies | 20 | For all outcomes considered (benefits or harms), present, for each study: 1) simple summary data for each intervention group, and 2) effect estimates and confidence intervals. Modified approaches may be needed to deal with information from larger networks. | 4-8, Figure 6 |
| Synthesis of results | 21 | Present results of each meta-analysis done, including confidence/confidence intervals. In larger networks, authors may focus on comparisons versus a particular comparator (e.g. placebo or standard care), with full findings presented in an appendix. League tables and forest plots may be considered to summarize pairwise comparisons. If additional summary measures were explored (such as treatment rankings), these should also be presented. | 4-9, Figure 4-6, |
| Exploration for inconsistency | S5 | Describe results from investigations of inconsistency. This may include such information as measures of model fit to compare consistency and inconsistency models, P values from statistical tests, or summary of inconsistency estimates from different parts of the treatment network. | 4,Supplementary Table 5 |
| Risk of bias across studies | 22 | Present results of any assessment of risk of bias across studies for the evidence base being studied. | Figure 2 |
| Results of additional analyses | 23 | Give results of additional analyses, if done (e.g., sensitivity or subgroup analyses, meta-regression analyses, alternative network geometries studied, alternative choice of prior distributions for Bayesian analyses, and so forth). | 10, Supplementary Table 6 |
|  |  |  |  |
| **DISCUSSION** |  |  |  |
| Summary of evidence | 24 | Summarize the main findings, including the strength of evidence for each main outcome; consider their relevance to key groups (e.g., healthcare providers, users, and policy-makers). | 10-11 |
| Limitations | 25 | Discuss limitations at study and outcome level (e.g., risk of bias), and at review level (e.g., incomplete retrieval of identified research, reporting bias). Comment on the validity of the assumptions, such as transitivity and consistency. Comment on any concerns regarding network geometry (e.g., avoidance of certain comparisons). | 12-13 |
| Conclusions | 26 | Provide a general interpretation of the results in the context of other evidence, and implications for future research. | 13 |
|  |  |  |  |
| **FUNDING** |  |  |  |
| Funding | 27 | Describe sources of funding for the systematic review and other support (e.g., supply of data); role of funders for the systematic review. This should also include information regarding whether funding has been received from manufacturers of treatments in the network and/or whether some of the authors are content experts with professional conflicts of interest that could affect use of treatments in the network. | 13 |

**Supplementary Table S2.** Search strategies for the network meta-analysis.

**2.1 Search strategy of China National Knowledge Infrastructure.**

**Search items**

(((SU %= '缺血性心肌病' OR SU %= '缺血性心脏病' OR SU %= '缺血性心力衰竭') OR ((SU %= '心力衰竭' OR SU %= '心功能不全' OR SU %= '心衰') AND (SU %= '冠心病' OR SU %= '冠状动脉粥样硬化性心脏病' OR SU %= '心肌梗死' OR SU %= '心梗'))) AND (TKA= '参附注射液' OR TKA= '参麦注射液' OR TKA= '生脉注射液' OR TKA= '黄芪注射液' OR TKA= '心脉隆注射液' OR TKA= '益气复脉注射液' OR TKA= '注射用益气复脉')) AND (TKA = '临床研究' OR TKA = '随机' OR FT = '随机' OR FT = '临床研究')

**2.2 Search strategy of Wanfang Database.**

**Search items**

(((主题:(缺血性心肌病) or 主题:(缺血性心脏病) or 主题:(缺血性心力衰竭)) or ((主题:(心力衰竭) or 主题:(心功能不全) or 主题:(心衰)) and (主题:(冠心病) or 主题:(冠状动脉粥样硬化性心脏病) or 主题:(心肌梗死) or 主题:(心梗)))) and (摘要:(参附注射液) or 摘要:(参麦注射液) or 摘要:(生脉注射液) or 摘要:(黄芪注射液) or 摘要:(心脉隆注射液) or 摘要:(注射用益气复脉) or 摘要:(益气复脉注射液))) and (摘要:(随机) or 摘要:(临床研究))

**2.3 Search strategy of Weipu Journal Database.**

**Search items**

((M=缺血性心肌病 OR M=缺血性心脏病 OR M=缺血性心力衰竭) OR ((M=心力衰竭 OR M=心功能不全 OR M=心衰) AND (M=冠心病 OR M=冠状动脉粥样硬化性心脏病 OR M=心梗 OR M=心肌梗死))) AND (R=参附注射液 OR R=参麦注射液 OR R=生脉注射液 OR R=黄芪注射液 OR R=心脉隆注射液 OR R=注射用益气复脉 OR R=益气复脉注射液)

**2.4 Search strategy of Chinese biomedical literature service system.**

**Search items**

(((( "缺血性心肌病"[摘要:智能] OR "缺血性心力衰竭"[摘要:智能] OR "缺血性心脏病"[摘要:智能]) AND( "参附注射液"[常用字段:智能] OR "参麦注射液"[常用字段:智能] OR "生脉注射液"[常用字段:智能] OR "黄芪注射液"[常用字段:智能] OR "心脉隆注射液"[常用字段:智能] OR "注射用益气复脉"[常用字段:智能] OR "益气复脉注射液"[常用字段:智能]))) OR ((( "心力衰竭"[常用字段:智能] OR "心功能不全"[常用字段:智能] OR "心衰"[常用字段:智能]) AND( "冠心病"[常用字段:智能] OR "冠状动脉粥样硬化性心脏病"[常用字段:智能] OR "心梗"[常用字段:智能] OR "心肌梗死"[常用字段:智能]) AND( "参附注射液"[常用字段:智能] OR "参麦注射液"[常用字段:智能] OR "生脉注射液"[常用字段:智能] OR "黄芪注射液"[常用字段:智能] OR "心脉隆注射液"[常用字段:智能] OR "注射用益气复脉"[常用字段:智能] OR "益气复脉注射液"[常用字段:智能]))))

**2.5 Search strategy of PubMed.**

| **No.** | **Search items** |
| --- | --- |
| #1 | Myocardial Ischemia[MeSH Terms] |
| #2 | "coronary artery insufficiency"[Title/Abstract] OR "coronary heart disease"[Title/Abstract] OR "coronary insufficiency"[Title/Abstract] OR "Disease*, Ischemic Heart"[Title/Abstract] OR "heart disease, coronary"[Title/Abstract] OR "heart disease, ischaemic"[Title/Abstract] OR "Heart Disease*, Ischemic"[Title/Abstract] OR "ischaemia heart disease"[Title/Abstract] OR "ischaemic cardiac disease"[Title/Abstract] OR "ischaemic cardial disease"[Title/Abstract] OR "ischaemic cardiopathy"[Title/Abstract] OR "ischaemic heart disease"[Title/Abstract] OR "ischemia heart disease"[Title/Abstract] OR "Ischemia*, Myocardial"[Title/Abstract] OR "ischemic cardiac disease"[Title/Abstract] OR "ischemic cardial disease"[Title/Abstract] OR "ischemic cardiopathy"[Title/Abstract] OR "ischemic heart disease*"[Title/Abstract] OR "Myocardial Ischemia*"[Title/Abstract] |
| #3 | #1 OR #2 |
| #4 | Heart Failure[MeSH Terms] |
| #5 | "backward failure, heart"[Title/Abstract] OR "cardiac backward failure"[Title/Abstract] OR "cardiac decompensation"[Title/Abstract] OR "cardiac failure"[Title/Abstract] OR "cardiac incompetence"[Title/Abstract] OR "cardiac insufficiency"[Title/Abstract] OR "cardiac stand still"[Title/Abstract] OR "cardial decompensation"[Title/Abstract] OR "cardial insufficiency"[Title/Abstract] OR "chronic heart failure"[Title/Abstract] OR "chronic heart insufficiency"[Title/Abstract] OR "decompensatio cordis"[Title/Abstract] OR "decompensation, heart"[Title/Abstract] OR "heart backward failure"[Title/Abstract] OR "heart decompensation"[Title/Abstract] OR "heart incompetence"[Title/Abstract] OR "heart insufficiency"[Title/Abstract] OR "insufficientia cardis"[Title/Abstract] OR "myocardial failure"[Title/Abstract] OR "myocardial insufficiency"[Title/Abstract] OR "heart failure"[Title/Abstract] |
| #6 | #4 OR #5 |
| #7 | Coronary Disease[MeSH Terms] |
| #8 | "Coronary Diseases"[Title/Abstract] OR "Disease, Coronary"[Title/Abstract] OR "Diseases, Coronary"[Title/Abstract] OR "Coronary Heart Disease"[Title/Abstract] OR "Coronary Heart Diseases"[Title/Abstract] OR "Disease, Coronary Heart"[Title/Abstract] OR "Diseases, Coronary Heart"[Title/Abstract] OR "Heart Disease, Coronary"[Title/Abstract] OR "Heart Diseases, Coronary"[Title/Abstract] |
| #9 | #7 OR #8 |
| #10 | Myocardial Infarction[MeSH Terms] |
| #11 | "cardiac infarct*"[Title/Abstract] OR "cardial infarct"[Title/Abstract] OR "Cardiovascular Stroke*"[Title/Abstract] OR "heart attack*"[Title/Abstract] OR "heart infarct"[Title/Abstract] OR "heart micro infarction"[Title/Abstract] OR "heart muscle infarction"[Title/Abstract] OR "myocardial infarct"[Title/Abstract] OR "Myocardial Infarcts"[Title/Abstract] OR "myocardium infarct"[Title/Abstract] OR "premonitory infarction sign"[Title/Abstract] OR "second heart attack"[Title/Abstract] OR "subendocardial infarction"[Title/Abstract] OR "transmural cardiac infarction"[Title/Abstract] OR "transmural heart infarction"[Title/Abstract] |
| #12 | #10 OR #11 |
| #13 | "xinmailong injection"[Title/Abstract] OR "shenfu injection"[Title/Abstract] OR "shenmai injection"[Title/Abstract] OR "shengmai injection"[Title/Abstract] OR "yiqifumai injection"[Title/Abstract] OR "yiqifumai lyophilized injection"[Title/Abstract] OR "huangqi injection"[Title/Abstract] OR "Astragalus Injection"[Title/Abstract] |
| #14 | "Controlled Clinical Trial" [Publication Type] OR "Randomized Controlled Trial"[Publication Type] OR "Equivalence Trial"[Publication Type] OR "Pragmatic Clinical Trial"[Publication Type]) OR "random*"[All Fields]) |
| #15 | (#3 OR (#6 AND (#9 OR #12))) AND #13 AND #14 |

**2.6 Search strategy of Embase.**

| **No.** | **Search items** |
| --- | --- |
| #1 | 'ischemic heart disease'/exp OR 'ischemic heart disease' |
| #2 | 'coronary artery insufficiency':ti,ab,kw OR 'coronary heart disease':ti,ab,kw OR 'coronary insufficiency':ti,ab,kw OR 'disease*, ischemic heart':ti,ab,kw OR 'heart disease, coronary':ti,ab,kw OR 'heart disease, ischaemic':ti,ab,kw OR 'heart disease*, ischemic':ti,ab,kw OR 'ischaemia heart disease':ti,ab,kw OR 'ischaemic cardiac disease':ti,ab,kw OR 'ischaemic cardial disease':ti,ab,kw OR 'ischaemic cardiopathy':ti,ab,kw OR 'ischaemic heart disease':ti,ab,kw OR 'ischemia heart disease':ti,ab,kw OR 'ischemia*, myocardial':ti,ab,kw OR 'ischemic cardiac disease':ti,ab,kw OR 'ischemic cardial disease':ti,ab,kw OR 'ischemic cardiopathy':ti,ab,kw OR 'ischemic heart disease*':ti,ab,kw OR 'myocardial ischemia*':ti,ab,kw |
| #3 | #1 OR #2 |
| #4 | 'heart failure'/exp OR 'backward failure, heart':ti,ab,kw OR 'cardiac backward failure':ti,ab,kw OR 'cardiac decompensation':ti,ab,kw OR 'cardiac failure':ti,ab,kw OR 'cardiac incompetence':ti,ab,kw OR 'cardiac insufficiency':ti,ab,kw OR 'cardiac stand still':ti,ab,kw OR 'cardial decompensation':ti,ab,kw OR 'cardial insufficiency':ti,ab,kw OR 'chronic heart failure':ti,ab,kw OR 'chronic heart insufficiency':ti,ab,kw OR 'decompensatio cordis':ti,ab,kw OR 'decompensation, heart':ti,ab,kw OR 'heart backward failure':ti,ab,kw OR 'heart decompensation':ti,ab,kw OR 'heart incompetence':ti,ab,kw OR 'heart insufficiency':ti,ab,kw OR 'insufficientia cardis':ti,ab,kw OR 'myocardial failure':ti,ab,kw OR 'myocardial insufficiency':ti,ab,kw OR 'heart failure':ti,ab,kw |
| #5 | 'coronary artery disease'/exp OR 'coronary diseases':ti,ab,kw OR 'disease, coronary':ti,ab,kw OR 'diseases, coronary':ti,ab,kw OR 'coronary heart disease':ti,ab,kw OR 'coronary heart diseases':ti,ab,kw OR 'disease, coronary heart':ti,ab,kw OR 'diseases, coronary heart':ti,ab,kw OR 'heart disease, coronary':ti,ab,kw OR 'heart diseases, coronary':ti,ab,kw |
| #6 | 'Myocardial Infarction':ti,ab,kw OR 'cardiac infarct*':ti,ab,kw OR 'cardial infarct':ti,ab,kw OR 'Cardiovascular Stroke*':ti,ab,kw OR 'heart attack*':ti,ab,kw OR 'heart infarct':ti,ab,kw OR 'heart micro infarction':ti,ab,kw OR 'heart muscle infarction':ti,ab,kw OR 'myocardial infarct':ti,ab,kw OR 'Myocardial Infarcts':ti,ab,kw OR 'myocardium infarct':ti,ab,kw OR 'premonitory infarction sign':ti,ab,kw OR 'second heart attack':ti,ab,kw OR 'subendocardial infarction':ti,ab,kw OR 'transmural cardiac infarction':ti,ab,kw OR 'transmural heart infarction':ti,ab,kw |
| #7 | #4 AND (#5 OR #6) |
| #8 | #3 OR #7 |
| #9 | 'xinmailong injection':ti,ab,kw OR 'shenfu injection':ti,ab,kw OR 'shenmai injection':ti,ab,kw OR 'shengmai injection':ti,ab,kw OR 'yiqifumai injection':ti,ab,kw OR 'yiqifumai lyophilized injection':ti,ab,kw OR 'huangqi injection':ti,ab,kw OR 'astragalus injection':ti,ab,kw |
| #10 | 'randomized controlled trial'/exp OR 'equivalence trial'/exp OR 'non-inferiority trial'/exp OR 'pragmatic trial'/exp OR 'superiority trial'/exp OR 'controlled clinical trial':it OR 'randomized controlled trial':it OR 'equivalence trial':it OR 'pragmatic clinical trial':it OR 'superiority trial':it OR 'non-inferiority trial':it OR random* |
| #11 | #8 AND #9 AND #10 |

**2.7 Search strategy of Web of Science.**

| **No.** | **Search items** |
| --- | --- |
| #1 | (TI=(("coronary artery insufficiency") OR ("coronary heart disease") OR ("coronary insufficiency") OR ("Disease*, Ischemic Heart") OR ("heart disease, coronary") OR ("heart disease, ischaemic") OR ("Heart Disease*, Ischemic") OR ("ischaemia heart disease") OR ("ischaemic cardiac disease") OR ("ischaemic cardial disease") OR ("ischaemic cardiopathy") OR ("ischaemic heart disease") OR ("ischemia heart disease") OR ("Ischemia*, Myocardial") OR ("ischemic cardiac disease") OR ("ischemic cardial disease") OR ("ischemic cardiopathy") OR ("ischemic heart disease*") OR ("Myocardial Ischemia*")) OR AB=(("coronary artery insufficiency") OR ("coronary heart disease") OR ("coronary insufficiency") OR ("Disease*, Ischemic Heart") OR ("heart disease, coronary") OR ("heart disease, ischaemic") OR ("Heart Disease*, Ischemic") OR ("ischaemia heart disease") OR ("ischaemic cardiac disease") OR ("ischaemic cardial disease") OR ("ischaemic cardiopathy") OR ("ischaemic heart disease") OR ("ischemia heart disease") OR ("Ischemia*, Myocardial") OR ("ischemic cardiac disease") OR ("ischemic cardial disease") OR ("ischemic cardiopathy") OR ("ischemic heart disease*") OR ("Myocardial Ischemia*")) OR AK=(("coronary artery insufficiency") OR ("coronary heart disease") OR ("coronary insufficiency") OR ("Disease*, Ischemic Heart") OR ("heart disease, coronary") OR ("heart disease, ischaemic") OR ("Heart Disease*, Ischemic") OR ("ischaemia heart disease") OR ("ischaemic cardiac disease") OR ("ischaemic cardial disease") OR ("ischaemic cardiopathy") OR ("ischaemic heart disease") OR ("ischemia heart disease") OR ("Ischemia*, Myocardial") OR ("ischemic cardiac disease") OR ("ischemic cardial disease") OR ("ischemic cardiopathy") OR ("ischemic heart disease*") OR ("Myocardial Ischemia*"))) |
| #2 | ((TI=(("backward failure, heart") OR ("cardiac backward failure") OR ("cardiac decompensation") OR ("cardiac failure") OR ("cardiac incompetence") OR ("cardiac insufficiency") OR ("cardiac stand still") OR ("cardial decompensation") OR ("cardial insufficiency") OR ("chronic heart failure") OR ("chronic heart insufficiency") OR ("decompensatio cordis") OR ("decompensation, heart") OR ("heart backward failure") OR ("heart decompensation") OR ("heart incompetence") OR ("heart insufficiency") OR ("insufficientia cardis") OR ("myocardial failure") OR ("myocardial insufficiency") OR ("heart failure")) OR AB=(("backward failure, heart") OR ("cardiac backward failure") OR ("cardiac decompensation") OR ("cardiac failure") OR ("cardiac incompetence") OR ("cardiac insufficiency") OR ("cardiac stand still") OR ("cardial decompensation") OR ("cardial insufficiency") OR ("chronic heart failure") OR ("chronic heart insufficiency") OR ("decompensatio cordis") OR ("decompensation, heart") OR ("heart backward failure") OR ("heart decompensation") OR ("heart incompetence") OR ("heart insufficiency") OR ("insufficientia cardis") OR ("myocardial failure") OR ("myocardial insufficiency") OR ("heart failure")) OR AK=(("backward failure, heart") OR ("cardiac backward failure") OR ("cardiac decompensation") OR ("cardiac failure") OR ("cardiac incompetence") OR ("cardiac insufficiency") OR ("cardiac stand still") OR ("cardial decompensation") OR ("cardial insufficiency") OR ("chronic heart failure") OR ("chronic heart insufficiency") OR ("decompensatio cordis") OR ("decompensation, heart") OR ("heart backward failure") OR ("heart decompensation") OR ("heart incompetence") OR ("heart insufficiency") OR ("insufficientia cardis") OR ("myocardial failure") OR ("myocardial insufficiency") OR ("heart failure"))) AND (TI=(("Coronary Diseases") OR ("Disease, Coronary") OR ("Diseases, Coronary") OR ("Coronary Heart Disease") OR ("Coronary Heart Diseases") OR ("Disease, Coronary Heart") OR ("Diseases, Coronary Heart") OR ("Heart Disease, Coronary") OR ("Heart Diseases, Coronary")) OR AB=(("Coronary Diseases") OR ("Disease, Coronary") OR ("Diseases, Coronary") OR ("Coronary Heart Disease") OR ("Coronary Heart Diseases") OR ("Disease, Coronary Heart") OR ("Diseases, Coronary Heart") OR ("Heart Disease, Coronary") OR ("Heart Diseases, Coronary")) OR AK=(("Coronary Diseases") OR ("Disease, Coronary") OR ("Diseases, Coronary") OR ("Coronary Heart Disease") OR ("Coronary Heart Diseases") OR ("Disease, Coronary Heart") OR ("Diseases, Coronary Heart") OR ("Heart Disease, Coronary") OR ("Heart Diseases, Coronary")))) |
| #3 | #1 OR #2 |
| #4 | (TI=(("xinmailong injection") OR ("shenfu injection") OR ("shenmai injection") OR ("shengmai injection") OR ("yiqifumai injection") OR ("yiqifumai lyophilized injection") OR ("huangqi injection") OR ("Astragalus Injection")) OR AB=(("xinmailong injection") OR ("shenfu injection") OR ("shenmai injection") OR ("shengmai injection") OR ("yiqifumai injection") OR ("yiqifumai lyophilized injection") OR ("huangqi injection") OR ("Astragalus Injection")) OR AK=(("xinmailong injection") OR ("shenfu injection") OR ("shenmai injection") OR ("shengmai injection") OR ("yiqifumai injection") OR ("yiqifumai lyophilized injection") OR ("huangqi injection") OR ("Astragalus Injection"))) |
| #5 | TS=(random*controlled trial OR random* OR placebo） |
| #6 | #3 AND #4 AND #5 |

**2.8 Search strategy of Cochrane Library.**

| **No.** | **Search items** |
| --- | --- |
| #1 | MeSH descriptor: [Myocardial Ischemia] explode all trees |
| #2 | ('coronary artery insufficiency' OR 'coronary heart disease' OR 'coronary insufficiency' OR 'Disease*, Ischemic Heart' OR 'heart disease, coronary' OR 'heart disease, ischaemic' OR 'Heart Disease*, Ischemic' OR 'ischaemia heart disease' OR 'ischaemic cardiac disease' OR 'ischaemic cardial disease' OR 'ischaemic cardiopathy' OR 'ischaemic heart disease' OR 'ischemia heart disease' OR 'Ischemia*, Myocardial' OR 'ischemic cardiac disease' OR 'ischemic cardial disease' OR 'ischemic cardiopathy' OR 'ischemic heart disease*' OR 'Myocardial Ischemia*'):ti,ab,kw |
| #3 | #1 OR #2 |
| #4 | MeSH descriptor: [Heart Failure, Systolic] explode all trees |
| #5 | ('backward failure, heart' OR 'cardiac backward failure' OR 'cardiac decompensation' OR 'cardiac failure' OR 'cardiac incompetence' OR 'cardiac insufficiency' OR 'cardiac stand still' OR 'cardial decompensation' OR 'cardial insufficiency' OR 'chronic heart failure' OR 'chronic heart insufficiency' OR 'decompensatio cordis' OR 'decompensation, heart' OR 'heart backward failure' OR 'heart decompensation' OR 'heart incompetence' OR 'heart insufficiency' OR 'insufficientia cardis' OR 'myocardial failure' OR 'myocardial insufficiency' OR 'heart failure'):ti,ab,kw |
| #6 | #4 OR #5 |
| #7 | MeSH descriptor: [Coronary Disease] explode all trees |
| #8 | ('Coronary Diseases' OR 'Disease, Coronary' OR 'Diseases, Coronary' OR 'Coronary Heart Disease' OR 'Coronary Heart Diseases' OR 'Disease, Coronary Heart' OR 'Diseases, Coronary Heart' OR 'Heart Disease, Coronary' OR 'Heart Diseases, Coronary'):ti,ab,kw |
| #9 | #7 OR #8 |
| #10 | MeSH descriptor: [Myocardial Infarction] explode all trees |
| #11 | ('cardiac infarct*' OR 'cardial infarct' OR 'Cardiovascular Stroke*' OR 'heart attack*' OR 'heart infarct' OR 'heart micro infarction' OR 'heart muscle infarction' OR 'myocardial infarct' OR 'Myocardial Infarcts' OR 'myocardium infarct' OR 'premonitory infarction sign' OR 'second heart attack' OR 'subendocardial infarction' OR 'transmural cardiac infarction' OR 'transmural heart infarction'):ti,ab,kw |
| #12 | #10 OR #11 |
| #13 | ('xinmailong injection' OR 'shenfu injection' OR 'shenmai injection' OR 'shengmai injection' OR 'yiqifumai injection' OR 'yiqifumai lyophilized injection' OR 'huangqi injection' OR 'Astragalus Injection'):ti,ab,kw |
| #14 | (Randomized Controlled Trial OR Controlled Clinical Trial OR Equivalence Trial OR Pragmatic Clinical Trial):pt OR (random*):ti,ab,kw |
| #15 | (#3 OR (#6 AND (#9 OR #12))) AND #13 AND #14 |

**Supplementary Table S3.** Basic characteristics of the 154 randomized controlled trials included in the network meta-analysis.

| **Study ID** | **n (C/T)** | **Sex, M/F (C/T)** | **Age (C/T), years** | **Intervention** | **Control** | **Duration, days** | **Follow-up** | **Outcomes** |
| --- | --- | --- | --- | --- | --- | --- | --- | --- |
| HanYX2018 | 30/30 | 19/11 / 18/12 | 57.21±6.25 / 57.26±6.34 | SFI 40ml qd | CT | 7 | NR | 4 |
| ZengY2009 | 23/28 | 14/9 / 16/11 | 72.7 / 74.3 | SFI 50ml qd | CT | 7 | NR | 1,5 |
| XuWJ2014 | 40/45 | 23/17 / 20/25 | 68.00±14.60 / 70.00±11.30 | SFI 80ml qd | CT | 10 | NR | 2,4,5 |
| TanHT2015 | 32/32 | 21/11 / 20/12 | 55.1±5.2 / 55.3±6.3 | SFI 60ml qd | CT | 14 | NR | 1,2,5,6 |
| LuoB2015 | 24/24 | 18/9 / 14/10 | 50.9±12.5 / 53.4±11.7 | SFI 50ml qd | CT | 7 | NR | 2,3,5 |
| HuangMX2019 | 52/52 | 32/20 / 36/16 | 72.4±8.2 / 72.6±12.1 | SFI 50ml qd | CT | 14 | NR | 2,5 |
| YaoDJ2017 | 37/37 | 26/11 / 24/13 | 55.78±6.7 / 55.42±6.6 | SFI 60ml qd | CT | 14 | NR | 11,5 |
| ChangJJ2012 | 50/50 | 32/18 / 34/16 | 69.0±3.55 / 68.5±4.10 | SFI 60ml qd | CT | 15 | NR | 1,5 |
| WuHJ2009 | 29/33 | 14/15 / 17/16 | 73.59±6.96 / 71.48±5.78 | SFI 50ml qd | CT | 14 | NR | 1,2,3,5,6 |
| XiuGX2015 | 25/23 | 12/13 / 13/10 | 63.4±9.8 / 65.5±10.1 | SFI 50ml qd | CT | 14 | NR | 2,3,5,6 |
| ZongB2014 | 53/52 | 33/20 / 29/23 | 65.31±11.37 / 65.32±12.12 | SFI 60ml qd | CT | 14 | NR | 1,2,3 |
| ChenZC2020 | 50/50 | 28/22 / 30/20 | 59.81±6.75 / 60.13±6.74 | SFI 60ml qd | CT | 14 | NR | 5,7 |
| LiXD2019 | 40/40 | 20/20 / 28/12 | 61.58±5.69 / 60.12±5.34 | SFI 40ml qd | CT | 90 | NR | 1,2,3,5,6 |
| YuLJ2012 | 31/31 | 32/30 / 32/30 | 68.2 / 68.2 | SFI 40ml qd | CT | 30 | NR | 1,2,5 |
| LiZC2018 | 30/30 | 19/11 / 16/14 | 65.2±11.3 / 63.8±12.8 | SFI 60ml qd | CT | 14 | NR | 5 |
| ZhouWJ2020 | 41/41 | 25/16 / 24/17 | 68.70±2.42 / 68.62±2.47 | SFI 50ml qd | CT | 14 | NR | 2,4 |
| WangH2018 | 25/25 | 15/10 / 16/9 | 53.6±11.8 / 54.0±11.1 | SFI 40ml qd | CT | 7 | NR | 1,4 |
| GuoJJ2006 | 26/36 | 15/11 / 20/16 | 60.5±9.6 / 62.2±11.3 | SFI 60ml qd | CT | 14 | NR | 1,3,5,6,7 |
| LiN2013 | 90/90 | 69/21 / 67/23 | 61±11 / 62±11 | SFI 60ml qd | CT | 15 | NR | 1,3,5 |
| TangJL2018 | 34/34 | 19/15 / 16/18 | 48.5±3.7 / 47.1±3.2 | SFI 100ml qd | CT | 14 | NR | 1,5,6 |
| XuW2015 | 34/33 | 18/16 / 16/17 | 69.32±9.13 / 71.24±7.17 | SFI 60ml qd | CT | 7 | NR | 1,2,4 |
| LiangCH2019 | 30/30 | 15/15 / 16/14 | 70.22±1.55 / 71.55±2.46 | SFI 50ml qd | CT | 10 | NR | 2,4,5 |
| WuHY2006 | 40/40 | 22/12 / 27/13 | 66.0±4.35 / 68.0±3.65 | SFI 50ml qd | CT | 10 | NR | 1,5 |
| WuSH2016 | 60/60 | 44/76 / 44/76 | 82.5±10 / 82.5±10 | SFI 60ml qd | CT | 20 | NR | 4,5 |
| SuXL2019 | 36/36 | 21/15 / 23/13 | 53.2±10.73 / 52.3±11.35 | SFI 50ml qd | CT | 14 | NR | 5,6 |
| WangH2016 | 30/26 | 16/14 / 13/13 | 70.23±1.56 / 71.56±2.47 | SFI 60ml qd | CT | 10 | NR | 1,2,4 |
| HuYH2009 | 32/31 | 12/20 / 12/19 | 76.43±6.80 / 72.94±7.58 | SFI 40ml qd | CT | 7 | NR | 5 |
| HeYL2016 | 45/45 | 30/15 / 29/16 | 62.3±7.8 / 61.4±8.3 | SFI 60ml qd | CT | 14 | NR | 3,5 |
| YuXY2014 | 32/35 | 41/26 / 41/26 | 58.3±2.3 / 58.3±2.3 | SFI 40ml qd | CT | 30 | NR | 1,2,5 |
| XuZQ2011 | 33/37 | 22/11 / 25/12 | 74.3±11.5 / 75.8±12.3 | SFI 50ml qd | CT | 14 | NR | 1,5 |
| GuoL2013 | 30/30 | 22/8 / 20/10 | 38-92 / 40-95 | SFI 50ml qd | CT | 14 | NR | 1 |
| ZhangYH2019 | 32/33 | 21/11 / 20/13 | 18-84 / 20-85 | SFI 60ml qd | CT | 10 | NR | 3,5,6,7 |
| LiWD2015 | 32/32 | 21/11 / 22/10 | 63.2±11.5 / 63.5±11.2 | SFI 60ml bid | CT | 14 | NR | 3,5 |
| MaXG2019 | 55/55 | 31/24 / 32/23 | 59.12±4.22 / 59.14±4.21 | SFI 60ml bid | CT | 14 | NR | 1,2,5 |
| ZhangZX2018 | 38/38 | 20/18 / 21/17 | 63.91±5.86 / 63.32±1.78 | SFI 50ml qd | CT | 28 | NR | 1,5 |
| GuoMY2014 | 40/40 | 24/16 / 22/18 | 71.2±14.6 / 73.7±16.2 | SFI 40ml bid | CT | 14 | NR | 1,5 |
| WangSN2021 | 32/33 | 22/10 / 21/12 | 72±13.6 / 73±12.8) | SFI 50ml qd | CT | 10 | NR | 1,3,5 |
| XuWD2019 | 45/45 | 23/22 / 25/20 | 64.3±5.3 / 65.1±5.5 | SFI 60ml qd | CT | 14 | NR | 1,3,5 |
| HanYC2018 | 30/30 | 15/15 / 15/15 | 57.43±7.92 / 55.53±6.73 | SFI 60ml qd | CT | 14 | NR | 1,3,5 |
| ZhangDY2012 | 39/39 | 24/15 / 24/15 | 61±13 / 61±12 | SFI 100ml qd | CT | 12 | NR | 1,3,5 |
| YangSQ2014 | 40/40 | 29/111 / 27/13 | 71.1±4.2 / 70.4±5.2 | SFI 50ml bid | CT | 10 | NR | 5 |
| WangXH2015 | 30/30 | 14/16 / 15/15 | 74±5 / 75±6 | SFI 1mg/kg·d | CT | 15 | NR | 3,5,6 |
| ShanY2019 | 46/46 | 25/21 / 27/19 | 71.46±5.17 / 71.35±5.24 | SMI 40ml qd | CT | 14 | NR | 2,3,5 |
| ZhangGX2018 | 32/32 | 18/14 / 17/115 | 63.80±2.69 / 63.75±2.73 | SMI 60ml qd | CT | 28 | 1年 | 1,2,5,6 |
| WeiYX2016 | 40/40 | 23/17 / 25/15 | 61.5±5.8 / 64.2±4.7 | SMI 60ml qd | CT | 7 | NR | 1,2,5 |
| YangQF2023 | 39/39 | 22/17 / 21/18 | 55.36±7.15 / 55.67±7.24 | SMI 30ml qd | CT | 90 | NR | 1,2,4,5,6 |
| SunMQ2015 | 58/59 | 32/26 / 34/25 | 63.4±8.7 / 65.6±8.4 | SMI 50ml qd | CT | 28 | NR | 2,5 |
| LiuXK2015 | 31/31 | 41/25 / 41/25 | 63.12±2.34 / 63.12±2.34 | SMI 60ml qd | CT | 28 | NR | 1,2,5 |
| WangXT2014 | 90/90 | 98/82 / 98/82 | 60.2 / 60.2 | SMI 40ml qd | CT | 15 | NR | 1 |
| DongWM2010 | 35/35 | 19/16 / 20/15 | 52-74 / 50-76 | SMI 50ml qd | CT | 30 | NR | 5 |
| ZhaoY2016 | 105/105 | 61/44 / 59/46 | 60.2±10.6 / 61.8±9.5 | SMI 50ml qd | CT | 14 | NR | 1,2,3,5,6 |
| LiHJ2011 | 43/43 | 54/32 / 54/32 | 62±8 / 62±8 | SMI 60ml qd | CT | 28 | NR | 1,2 |
| ZhengY2016 | 34/34 | 21/13 / 19/15 | 66.5±4.7 / 64.3±4.6 | SMI 60ml qd | CT | 28 | NR | 2 |
| ZhaoLM2012 | 35/35 | 27/8 / 26/9 | 67.3±9.0 / 66.8±8.4 | SMI 60ml qd | CT | 7 | NR | 1 |
| ZhaoY2011 | 53/53 | 27/26 / 28/25 | 54.1 / 53.3 | SMI 60ml qd | CT | 15 | NR | 4,5 |
| CaoFD2012 | 60/60 | 85/25 / 85/25 | 54 / 54 | SMI 100ml qd | CT | 14 | NR | 1,2 |
| XiaoML2018 | 40/40 | 23/17 / 22/18 | 65.43±4.38 / 65.45±4.40 | SMI 50ml qd | CT | 15 | NR | 1,2 |
| YangZG2012 | 30/30 | 18/12 / 17/13 | 67±2.56 / 65.5±3.29 | SMI 100ml qd | CT | 7 | NR | 1,2 |
| YuQ2024 | 59/59 | 31/28 / 34/25 | 59.16±5.12 / 58.18±5.46 | SMI 50ml qd | CT | 30 | NR | 1,2,3,5,6 |
| JiangHP2007 | 30/30 | 16/14 / 19/11 | 61 / 67 | SMI 50ml qd | CT | 14 | NR | 1,2,5 |
| PanH2014 | 75/75 | 44/31 / 43/32 | 67.35±13.45 / 66.35±13.26 | SMI 100ml qd | CT | 14 | NR | 1,2,5,7 |
| YangJW2017 | 38/38 | 25/13 / 21/17 | 35.4±6.7 / 36.8±5.4 | SMI 50ml qd | CT | 14 | NR | 1,3,5,6 |
| LiuWS2019 | 63/63 | 26/37 / 29/24 | 67.08±6.78 / 66.57±6.81 | SMI 20ml qd | CT | 14 | NR | 1,3,5,6 |
| HuangBF2018 | 49/49 | 26/23 / 29/20 | 66.79±6.90 / 66.10±6.93 | SMI 20ml qd | CT | 14 | NR | 1,3 |
| GengHB2018 | 42/42 | 22/20 / 23/19 | 62.38±3.26 / 63.62±3.17 | SMI 60ml qd | CT | 14 | NR | 1,2,3,5,6 |
| PingP2021 | 45/45 | 20/25 / 24/21 | 52.23±4.12 / 53.12±3.99 | SMI 50ml qd | CT | 28 | NR | 1,5 |
| CaoXX2021 | 49/49 | - / - | 53.45±2.75 / 54.05±1.97 | SMI 50ml qd | CT | 28 | NR | 1,5 |
| LiXX2018 | 56/56 | 31/25 / 30/26 | 60.32±2.16 / 60.11±2.08 | SMI 50ml qd | CT | 30 | NR | 1,2,3,5,6,7 |
| FengX2016 | 75/75 | 39/36 / 40/35 | 61.11±2.58 / 61.35±2.62 | SMI 50ml qd | CT | 14 | NR | 1,5,6,7 |
| CaiDL2016 | 32/32 | 20/12 / 21/11 | 65.2±7.4 / 65.1±7.5 | SMI 50ml qd | CT | 14 | NR | 1,2,3,5 |
| YuSC2017 | 66/67 | 56/10 / 64/3 | 64±9 / 61±11 | SMI 50ml qd | CT+placebo | 28 | 12月 | 3,5,7 |
| WangQX2004 | 30/30 | 45/15 / 45/15 | 59±6 / 59±6 | SMI 40ml qd | CT | 14 | NR | 1 |
| LiJ2016 | 60/60 | 35/25 / 37/23 | 59.32±8.35 / 61.32±8.61 | SMI 100ml qd | CT | 14 | NR | 3,5 |
| WangJZ2013 | 42/45 | 26/16 / 27/18 | 66.8±2.4 / 67.4±2.6 | SMI 50ml qd | CT | 56 | NR | 2,5,6 |
| SuJ2012 | 37/37 | 43/31 / 43/31 | 61.32±2.54 / 61.32±2.54 | SMI 50ml qd | CT | 28 | NR | 2,5 |
| XianSS2016 | 114/114 | 66/48 / 71/43 | 68.12±8.88 / 68.95±9.91 | SMI 100ml qd | CT | 7 | NR | 2,5,7 |
| HongCX2009 | 26/24 | 19/7 / 19/5 | 67.21 / 68.36 | SHMI 50ml qd | CT | 10 | NR | 1,5 |
| GongLF2001 | 107/120 | 72/35 / 75/45 | 63.2 / 64.7 | SHMI 60ml qd | CT | 14 | NR | 1,2,5 |
| WuFX2009 | 23/27 | 12/11 / 15/12 | 57 / 55 | SHMI 60ml qd | CT | 14 | NR | 5 |
| DongW2010 | 40/42 | 26/14 / 29/13 | 64.0±6.2 / 65.0±7.8 | SHMI 50ml qd | CT | 14 | NR | 1 |
| ZhaoHJ2011 | 36/36 | 44/28 / 44/28 | 60.6±10.4 / 60.6±10.4 | SHMI 50ml qd | CT | 21 | NR | 1,2,5 |
| YangDF2007 | 34/28 | 36/26 / 36/26 | 45-80 / 45-80 | SHMI 60ml qd | CT | 10 | NR | 1,5,6 |
| XuLJ2009 | 39/39 | - / - | 60.9±11.7 / 60.9±11.7 | SHMI 40ml qd | CT | 14 | NR | 1,5,6 |
| LianYJ2018 | 43/43 | 28/15 / 29/14 | 62.27±5.25 / 62.34±5.28 | SHMI 50ml qd | CT | 7 | NR | 1,5,6 |
| HuangYM1999 | 40/40 | 23/17 / 22/18 | 44-80 / 42-81 | SHMI 20ml qd | CT | 14 | NR | 1,2 |
| HuangGN2012 | 35/35 | 26/9 / 28/7 | 60.2±8.1 / 58.7±7.6 | SHMI 40ml qd | CT | 7 | NR | 1,3,5,6 |
| RongW2018 | 32/32 | 20/12 / 18/14 | 62.1±2.5 / 62.3±2.4 | SHMI 40ml qd | CT | 7 | NR | 1,5,6 |
| XinQ2012 | 28/28 | 16/12 / 15/13 | 59.6±9.2 / 58.9±8.7 | SHMI 40ml qd | CT | 7 | NR | 1,2,5,6 |
| DingJR2012 | 40/40 | 15/25 / 18/22 | 72.56±5.64 / 70.74±6.43 | SHMI 40ml qd | CT | 7 | NR | 2,5 |
| WangWL2006 | 40/40 | 50/30 / 50/30 | 65±8 / 65±8 | SHMI 40ml qd | CT | 14 | NR | 3,5 |
| WuQF2020 | 41/41 | 26/15 / 27/14 | 63.45±2.73 / 63.74±2.66 | SHMI 40ml qd | CT | 7 | NR | 4,5,6 |
| ZhaoYH2008 | 39/39 | - / - | 60.9~11.9 / 61.2~9.9 | SHMI 40ml qd | CT | 14 | NR | 1,2,5 |
| ZhouHH2015 | 50/50 | 26/24 / 27/23 | 62.1±8.7 / 62.3±8.5 | SHMI 60ml qd | CT | 15 | NR | 1 |
| XuanN2015 | 100/100 | 52/48 / 54/46 | 62.1±8.7 / 62.3±8.5 | SHMI 60ml qd | CT | 15 | NR | 1 |
| ShangYN2015 | 52/52 | 63/41 / 63/41 | 62.1±8.7 / 62.1±8.7 | SHMI 60ml qd | CT | 15 | NR | 1 |
| ZhangS2015 | 53/53 | 31/22 / 30/23 | 65.9±5.3 / 67.2±5.8 | HQI 20ml qd | CT | 14 | NR | 1,2,3,5,7 |
| CaiY2013 | 60/60 | 29/31 / 28/32 | 62.35±10.5 / 62.36±10.54 | HQI 20ml qd | CT | 14 | NR | 1,2,5,6 |
| ChengJ2015 | 30/50 | 12/18 / 28/22 | 63.4±1.6 / 67.6±2.7 | HQI 20ml qd | CT | 10 | NR | 1,5 |
| LuZT2006 | 39/39 | 42/36 / 42/36 | - / - | HQI 40ml qd | CT | 14 | NR | 1,5 |
| WangXN2003 | 31/35 | 24/7 / 26/9 | 60.6±10.6 / 60.9±11.7 | HQI 40ml qd | CT | 14 | NR | 2,5 |
| LinH2010 | 32/32 | 1999/13 / 20/12 | 72.0±12.5 / 71.6±4.5 | HQI 60ml qd | CT | 21 | NR | 1,2 |
| GuX2003 | 66/68 | 70/64 / 70/64 | 55.8 / 55.8 | HQI 40ml qd | CT | 28 | NR | 11,5 |
| ZhouZM2002 | 47/56 | 35/12 / 37/19 | 59.4±6.3 / 61.3±5.7 | HQI 60ml qd | CT | 21 | NR | 1,5 |
| LiW2017 | 50/50 | - / - | 55.27±6.75 / 54.92±5.83 | XMLI 5mg/kg bid | CT | 14 | NR | 1,3 |
| SuRY2020 | 51/51 | 25/26 / 23/28 | 47.8±4.3 / 48.5±4.1 | XMLI 5mg/kg bid | CT | 10 | NR | 1,2,3,5,6 |
| LiangT2014 | 32/32 | 19/13 / 20/12 | 59.8±9.5 / 60.3±7.5 | XMLI 5mg/kg bid | CT | 14 | NR | 1,2,3,5 |
| YanRY2019 | 61/61 | 39/22 / 37/24 | 86.35±7.67 / 87.12±6.98 | XMLI 6ml bid | CT | 10 | NR | 3,5 |
| ShenWY2017 | 58/58 | 36/22 / 34/24 | 61.6±7.8 / 62.8±7.1 | XMLI 4ml bid | CT | 14 | NR | 1,4,5,7 |
| WuZF2017 | 42/48 | 31/11 / 28/20 | 56.13±4.87 / 54.05±3.96 | XMLI 5mg/kg bid | CT | 10 | NR | 3,5,7 |
| TangJJ2024 | 75/75 | 40/35 / 43/32 | 44.88±2.14 / 44.55±2.45 | XMLI 5mg/kg bid | CT | 7 | NR | 1,2,4,5,6,7 |
| YangXQ2015 | 34/33 | - / - | - / - | XMLI 5mg/kg bid | CT | 10 | NR | 1,2,3 |
| YuanXY2019 | 43/43 | 29/14 / 27/16 | 68.02±9.35 / 67.85±8.97 | XMLI 6ml bid | CT | 7 | NR | 3,5 |
| ZhangXH2013 | 30/30 | 21/9 / 19/11 | - / - | XMLI 5mg/kg bid | CT | 10 | NR | 5 |
| LiTT2016 | 23/23 | 28/18 / 28/18 | 56.87±5.43 / 56.87±5.43 | XMLI 6ml bid | CT | 14 | NR | 1,3,5 |
| SongFF2022 | 60/60 | 35/25 / 38/22 | 68.3±4.3 / 69.1±4.0 | XMLI 6ml bid | CT | 7 | NR | 4,5,6 |
| WuTK2015 | 50/50 | 29/21 / 24/26 | 71±7.1 / 70±7.5 | XMLI 5mg/kg bid | CT | 10 | NR | 1,3 |
| SongXX2016 | 42/42 | 31/31 / 20/22 | 59.63±4.43 / 59.65±4.46 | XMLI 5mg/kg bid | CT | 10 | NR | 1,2,3,5 |
| HuangXC2022 | 50/50 | 30/20 / 26/24 | 60.19±3.67 / 60.24±3.69 | XMLI 5mg/kg bid | CT | 14 | NR | 1,2,5 |
| SongXF2023 | 36/36 | 19/17 / 21/15 | 61.40±4.19 / 60.20±4.17 | XMLI 5mg/kg bid | CT | 14 | NR | 4,5,6 |
| LiangSY2022 | 28/28 | 15/13 / 16/12 | 63.24±3.33 / 63.27±3.24 | XMLI 5mg/kg bid | CT | 7 | NR | 1,2,4 |
| XuF2019 | 51/57 | 24/27 / 25/32 | 60.28±6.41 / 61.39±5.73 | XMLI 5mg/kg bid | CT | 7 | NR | 1,5 |
| KangXT2021 | 30/30 | 14/16 / 13/17 | 56.79±2.24 / 56.81±2.25 | XMLI 5mg/kg bid | CT | 14 | NR | 1,2,5 |
| ZhangXH2016 | 35/35 | 20/15 / 22/13 | - / - | XMLI 6ml bid | CT | 10 | NR | 4,5 |
| WeiXF2020 | 40/40 | 22/18 / 23/17 | 54.25±6.41 / 53.64±7.56 | XMLI 5mg/kg bid | CT | 7 | NR | 3 |
| ZhangJ2025 | 55/55 | 30/25 / 31/24 | 56.61±2.45 / 56.59±2.54 | XMLI 6ml bid | CT | 14 | NR | 1,2,4,5 |
| LiJF2016 | 50/50 | 34/16 / 37/13 | 84±5.1 / 85±4.6 | XMLI 100mg bid | CT | 14 | NR | 3,5 |
| YanXB2019 | 35/35 | 21/14 / 23/12 | 73.4±8.2 / 72.6±7.9 | XMLI 6ml bid | CT | 14 | NR | 1,2,3,5,6,7 |
| HanZ2018 | 80/80 | - / - | - / - | XMLI 6ml bid | CT | 10 | NR | 1 |
| ZhangXH2020 | 40/40 | 19/21 / 21/19 | 64.8±3.5 / 63.3±2.3 | XMLI 2ml bid | CT | 7 | NR | 1,4,5,7 |
| YangZM2021 | 63/63 | 38/25 / 36/27 | 70±5 / 70±5 | YQFMI 5.2g qd | CT | 14 | NR | 2,4,5,6 |
| ShaoN2022 | 40/40 | 22/18 / 24/16 | 57.4±5.77 / 57.52±5.85 | YQFMI 5.2g qd | CT | 28 | NR | 4,5,6,7 |
| MaXC2020 | 25/25 | 16/9 / 17/8 | 65.26±3.04 / 63.04±3.15 | YQFMI 5.2g qd | CT | 30 | NR | 5,6 |
| WangSL2019 | 49/49 | 33/16 / 32/17 | 79.01±10.67 / 75.96±13.64 | YQFMI 5.2g qd | CT | 14 | NR | 1,5,6 |
| XiaoJX2022 | 30/30 | 20/10 / 16/14 | 74.09±7.82 / 73.52±8.03 | YQFMI 5.2g qd | CT | 14 | NR | 1,4 |
| LiY2019 | 59/59 | 34/25 / 35/24 | 68.33±4.84 / 68.45±4.78 | YQFMI 5.2g qd | CT | 14 | NR | 2,4,5,6 |
| WangY2025 | 45/45 | 22/23 / 23/22 | 62.31±1.24 / 62.33±1.22 | YQFMI 5.2g qd | CT | 14 | NR | 1,2,5,6 |
| ZhuH2019 | 100/100 | 66/34 / 66/34 | 63.24±5.31 / 63.68±5.32 | YQFMI 5.2g qd | CT | 14 | NR | 1,2,5,6 |
| LiaoK2022 | 40/40 | 22/18 / 19/21 | 51.77±5.09 / 52.13±5.78 | YQFMI 5.2g qd | CT | 14 | NR | 1,2,4,5,6 |
| ChiZF2023 | 60/60 | 38/22 / 40/20 | 63.38±1.46 / 63.42±1.57 | YQFMI 3.9g qd | CT | 14 | NR | 1,2,5,6 |
| MaoJF2018 | 30/30 | 18/12 / 17/13 | 70.5±2.35 / 71.8±2.32 | YQFMI 5.2g qd | CT | 15 | NR | 1,4,5 |
| WangYP2025 | 45/45 | 24/21 / 25/20 | 60.92±2.64 / 61.01±2.68 | YQFMI 5.2g qd | CT | 14 | NR | 1,2,5 |
| YangYJ2016 | 30/30 | 23/7 / 22/8 | 70.8±3.40 / 70.7±3.28 | YQFMI 5.2g qd | CT | 14 | NR | 4,5 |
| WangJ2018 | 50/50 | 30/20 / 32/18 | 69.5±5.8 / 70.0±6.0 | YQFMI 5.2g qd | CT | 14 | NR | 1,2,4,5 |
| WangSJ2025 | 51/51 | 28/23 / 26/25 | 72.34±5.04 / 71.89±4.8 | YQFMI 5.2g qd | CT | 7 | NR | 1,4,5,6,7 |
| LiaoF2014 | 28/28 | 32/24 / 32/24 | 70-85 / 70-85 | YQFMI 2.6g qd | CT | 14 | NR | 4,5 |
| WangM2010 | 39/39 | 24/15 / 21/18 | 60.11±11.43 / 58.81±14.87 | YQFMI 2.6g qd | CT | 14 | NR | 5,6 |
| ZhouZL2017 | 33/33 | 37/29 / 37/29 | 65.92±1.21 / 65.92±1.21 | YQFMI 3.9g qd | CT | 14 | NR | 4,5,6,7 |
| DaiH2017 | 38/38 | 22/16 / 21/17 | 60.64±5.85 / 60.74±5.29 | YQFMI 5.2g qd | CT | 14 | NR | 4,5,6,7 |
| WangQ2024 | 60/61 | 38/22 / 36/25 | 67.02±10.15 / 66.1±8.41 | YQFMI 5.2g qd | CT | 7 | NR | 2,4 |
| YuZ2015 | 45/45 | 23/22 / 25/20 | 52 / 51.5 | YQFMI 2.6g qd | CT | 14 | NR | 1,5 |
| FengC2013 | 24/27 | - / - | 56-86 / 56-86 | YQFMI 5.2g qd | CT | 14 | NR | 1,3,5,6 |
| SunJH2015 | 60/60 | 44/16 / 43/17 | 58.2±4.8 / 58.4±4.7 | YQFMI 5.2g qd | CT | 14 | NR | 1,2,4,5 |
| YangCL2014 | 60/60 | - / - | 73.1±5.4 / 72.2±4.3 | YQFMI 5.2g qd | CT | 14 | NR | 1,4,5 |
| GongKW2023 | 35/35 | 16/19 / 18/17 | 63.57±5.73 / 62.83±5.58 | YQFMI 2.6g qd | CT | 10 | NR | 1,2,4,5 |

Abbreviations: ADR, adverse drug reaction; BNP, B-type natriuretic peptide; C, control group; CT, conventional treatment; LVEF, left ventricular ejection fraction; LVEDD, left ventricular end-diastolic dimension; NR, not reported; NT-proBNP, N-terminal pro-B-type natriuretic peptide; T, treatment group; 6MWT, 6-minute walk test. Outcome codes: 1, clinical effectiveness rate; 2, ADR; 3, BNP; 4, NT-proBNP; 5, LVEF; 6, LVED; 7, 6MWT.

**Supplementary Table S4.** Basic information on the six traditional Chinese medicine injections included in the analysis.

| **Number** | **Generic name** | **Chemical composition** | **Botanical name** |
| --- | --- | --- | --- |
| 1 | Shenfu injection | Ginsenosides and aconite alkaloids (extracts of red ginseng and processed aconite root) | *Panax ginseng C.A.Mey.; Aconitum carmichaelii Debeaux* |
| 2 | Shenmai injection | Ginsenosides and ophiopogonins | *Panax ginseng C.A.Mey.; Ophiopogon japonicus (Thunb.) Ker Gawl.* |
| 3 | Shengmai injection | Ginsenosides; ophiopogonins; Schisandra lignans | *Panax ginseng C.A.Mey.; Ophiopogon japonicus (Thunb.) Ker Gawl.; Schisandra chinensis (Turcz.) Baill.* |
| 4 | Huangqi injection/Astragalus Injection | Astragalosides (especially astragaloside IV), polysaccharides and flavonoids | *Astragalus membranaceus (Fisch.) Bunge.* |
| 5 | Xinmailong injection | Composite peptides; adenosine, inosine, protocatechuic acid and pyroglutamyl dipeptides | *Periplaneta americana L.* |
| 6 | Yiqifumai injection/Yiqifumai lyophilized injection | Triterpenoid, lignans; flavonoids | *Panax ginseng C.A.Mey.; Ophiopogon japonicus (Thunb.) Ker Gawl.; Schisandra chinensis (Turcz.) Baill.* |

**Supplementary Table S6.** Network geometry by outcomes.

| outcome | RCTs(n) | TCMIs(n) | Interventing nodes(n) | Common comparator | head-to-head TCMI trials(n) | Closed loops  (n) | Evidence for TCMI-vs-TCMI | network geometry |
| --- | --- | --- | --- | --- | --- | --- | --- | --- |
| CER | 103 | 6 | 7 | CT | 0 | 0 | indirect | CT-centered Star Network |
| ADRs | 65 | 6 | 7 | CT | 0 | 0 | indirect | CT-centered Star Network |
| BNP | 44 | 6 | 7 | CT | 0 | 0 | indirect | CT-centered Star Network |
| NT-proBNP | 35 | 5 | 6 | CT | 0 | 0 | indirect | CT-centered Star Network |
| LVEF | 124 | 6 | 7 | CT | 0 | 0 | indirect | CT-centered Star Network |
| LVEDD | 45 | 6 | 7 | CT | 0 | 0 | indirect | CT-centered Star Network |
| 6MWT | 17 | 5 | 6 | CT | 0 | 0 | indirect | CT-centered Star Network |

**Supplementary Table S6.** Sensitivity analyses using alternative pre–post correlation coefficients.

| Outcome | Treatment | R（primary，low，high） | Primary analysis, MD (95% CrI) | Lower-r sensitivity analysis, MD (95% CrI) | Higher-r sensitivity analysis, MD (95% CrI) | Rank (Primary / Lower r / Higher r) |
| --- | --- | --- | --- | --- | --- | --- |
| BNP | SFI+CT vs CT | 0.5，0.25，0.75 | -1.90(-2.76, -1.39) | -1.64(-2.43, -0.86) | -2.37(-3.37, -1.37) | 4/4/4 |
|  | SMI+CT vs CT |  | -2.79(-3.68, -1.91) | -2.37(-3.12, -1.62) | -3.60(-4.75, -2.45) | 2/2/2 |
|  | SHMI+CT vs CT |  | -5.45(-9.35, -1.55) | -4.63(-8.07, -1.18) | -6.98(-11.47, -2.48) | 1/1/1 |
|  | HQI+CT vs CT |  | 0.07(-0.31, 0.45) | 0.06(-0.32, 0.44) | 0.08(-0.31, 0.46) | 6/6/6 |
|  | XMLI+CT vs CT |  | -1.70(-2.26, -1.14) | -1.50(-2.03, -0.98) | -2.03(-2.65, -1.41) | 3/3/3 |
|  | YQFMI+CT vs CT |  | -0.25(-0.80, 0.31) | -0.21(-0.76, 0.35) | -0.33(-0.88, 0.23) | 5/5/5 |
|  | Overall |  | -2.17(-2.63, -1.72) | -1.87(-2.28, -1.46) | -2.73(-3.27, -2.19) |  |
| NT-pro BNP | SFI+CT vs CT |  | -1.30(-1.96, 0.63) | -1.18(-1.78, -0.57) | -1.62(-2.37, -0.88) | 3/3/3 |
|  | SMI+CT vs CT |  | -0.68(-1.28, -0.07) | -0.56(-1.05, -0.06) | -0.93(-1.78, -0.08) | 4/4/4 |
|  | SHMI+CT vs CT |  | -2.83(-3.44, -2.21) | -2.43(-3.00, -1.85) | -3.52(-4.22, -2.83) | 2/2/2 |
|  | XMLI+CT vs CT |  | -2.94(-4.04, -1.84) | -2.60(-3.56, -1.64) | -3.52(-4.88, -2.17) | 1/1/1 |
|  | YQFMI+CT vs CT |  | -1.81(-2.88, -0.74) | -1.45(-2.45, -0.45) | -2.52(-3.70, -1.34) | 5/5/5 |
|  | Overall |  | -1.97(-2.56, -1.38) | -1.69(-2.23, -1.15) | -2.52(-3.18, -1.86) |  |
| LVEF | SFI+CT vs CT | 0.8，0.5，0.9 | 5.43(4.16, 6.70) | 5.41(4.16, 6.66) | 5.43(4.13, 6.74) | 4/4/4 |
|  | SMI+CT vs CT |  | 9.61(6.78, 12.43) | 9.60(6.79, 12.41) | 9.61(6.75, 12.47) | 1/1/1 |
|  | SHMI+CT vs CT |  | 7.35(5.89, 8.81) | 7.20(5.68, 8.72) | 7.48(6.06, 8.90) | 2/2/2 |
|  | HQI+CT vs CT |  | 4.94(3.48, 6.40) | 4.94(3.50, 6.38) | 4.95(3.48, 6.42) | 5/5/5 |
|  | XMLI+CT vs CT |  | 5.86(3.79, 7.92) | 5.84(3.77, 7.92) | 5.86(3.83, 7.90) | 3/3/3 |
|  | YQFMI+CT vs CT |  | 3.74(1.59, 5.90) | 3.72(1.68, 5.76) | 3.75(1.49, 6.01) | 6/6/6 |
|  | Overall |  | 6.23(5.65, 6.80) | 6.19(5.60, 6.78) | 6.26(5.68, 6.83) |  |
| LVEDD | SFI+CT vs CT |  | -2.69(-7.1, 1.75) | -2.71(-7.23, 1.81) | -2.68(-6.99, 1.63) | 6/6/6 |
|  | SMI+CT vs CT |  | -6.37(-8.19, -4.54) | -6.35(-8.21, -4.49) | -6.37(-8.16, -4.58) | 2/2/2 |
|  | SHMI+CT vs CT |  | -3.12(-4.56, -1.67) | -3.25(-4.68, -1.82) | -3.01(-4.46, -1.56) | 5/5/5 |
|  | HQI+CT vs CT |  | -6.11(-7.29, -4.93) | -6.11(-7.74, -4.48) | -6.11(-6.98, -5.24) | 3/3/3 |
|  | XMLI+CT vs CT |  | -4.92(-6.39, -3.45) | -4.78(-6.28, -3.27) | -5.01(-6.44, -3.58) | 4/4/4 |
|  | YQFMI+CT vs CT |  | -8.74(-16.74, -0.74) | -8.75(-16.52, -0.98) | -8.73(-17.03, -0.43) | 1/1/1 |
|  | Overall |  | -5.79(-9.00, -2.58) | -5.81(-8.94, -2.67) | -5.78(-9.06, -2.50) |  |
| 6WMT | SFI+CT vs CT | 0.7，0.5，0.9 | 99.26(29.01, 169.51) | 99.27(28.14, 170.34) | 99.24(32.05, 166.42) | 1/1/1 |
|  | SMI+CT vs CT |  | 49.18(30.76, 67.60) | 48.62(29.87, 67.36) | 49.70(31.61, 67.80) | 3/3/3 |
|  | HQI+CT vs CT |  | 42.11(21.86, 62.36) | 42.11(20.38, 63.84) | 42.11(23.46, 60.76) | 5/5/5 |
|  | XMLI+CT vs CT |  | 42.46(20.71, 62.21) | 41.05(18.92, 63.17) | 44.62(23.42, 65.82) | 4/4/4 |
|  | YQFMI+CT vs CT |  | 50.45(42.08, 58.82) | 50.22(39.97, 60.46) | 52.55(45.03, 60.07) | 2/2/2 |
|  | Overall |  | 57.11(45.89, 68.34) | 56.54(44.85, 68.22) | 57.74(47.46, 68.01) |  |

**Supplementary Table S7.** Confidence ratings from CINeMA assessments.

| Comparison | N | Within-study bias | Reporting bias | Indirectness | Imprecision | Heterogeneity | Incoherence | Confidence rating |
| --- | --- | --- | --- | --- | --- | --- | --- | --- |
| CER | Mixed evidence | | | | | | | |
| CT:HQI | 7 | No concerns | No concerns | No concerns | No concerns | No concerns | Major concerns | Low |
| CT:SFI | 25 | No concerns | Some concerns | No concerns | No concerns | No concerns | Major concerns | Very low |
| CT:SHMI | 15 | No concerns | Some concerns | No concerns | No concerns | No concerns | Major concerns | Very low |
| CT:SMI | 24 | No concerns | No concerns | No concerns | No concerns | No concerns | Major concerns | Low |
| CT:XMLI | 17 | No concerns | No concerns | No concerns | No concerns | No concerns | Major concerns | Low |
| CT:YQFMI | 15 | No concerns | No concerns | No concerns | No concerns | No concerns | Major concerns | Low |
| CER | Indirect evidence | | | | | | | |
| HQI:SFI | 0 | No concerns | Some concerns | No concerns | Major concerns | No concerns | Major concerns | Very low |
| HQI:SHMI | 0 | No concerns | Some concerns | No concerns | Major concerns | No concerns | Major concerns | Very low |
| HQI:SMI | 0 | No concerns | No concerns | No concerns | Major concerns | No concerns | Major concerns | Low |
| HQI:XMLI | 0 | No concerns | No concerns | No concerns | Major concerns | No concerns | Major concerns | Low |
| HQI:YQFMI | 0 | No concerns | No concerns | No concerns | Some concerns | No concerns | Major concerns | Very low |
| SFI:SHMI | 0 | No concerns | Some concerns | No concerns | Major concerns | No concerns | Major concerns | Very low |
| SFI:SMI | 0 | No concerns | Some concerns | No concerns | Some concerns | No concerns | Major concerns | Very low |
| SFI:XMLI | 0 | No concerns | Some concerns | No concerns | Some concerns | No concerns | Major concerns | Very low |
| SFI:YQFMI | 0 | No concerns | No concerns | No concerns | Some concerns | No concerns | Major concerns | Low |
| SHMI:SMI | 0 | No concerns | Some concerns | No concerns | Major concerns | No concerns | Major concerns | Very low |
| SHMI:XMLI | 0 | No concerns | Some concerns | No concerns | Major concerns | No concerns | Major concerns | Very low |
| SHMI:YQFMI | 0 | No concerns | Some concerns | No concerns | Major concerns | No concerns | Major concerns | Very low |
| SMI:XMLI | 0 | No concerns | No concerns | No concerns | Major concerns | No concerns | Major concerns | Low |
| SMI:YQFMI | 0 | No concerns | No concerns | No concerns | Major concerns | No concerns | Major concerns | Low |
| XMLI:YQFMI | 0 | No concerns | No concerns | No concerns | Major concerns | No concerns | Major concerns | Low |
| ADR | Mixed evidence | | | | | | | |
| CT:HQI | 4 | No concerns | Some concerns | No concerns | Major concerns | No concerns | Major concerns | Very low |
| CT:SFI | 14 | No concerns | Some concerns | No concerns | Some concerns | No concerns | Major concerns | Very low |
| CT:SHMI | 6 | No concerns | Some concerns | No concerns | Major concerns | No concerns | Major concerns | Very low |
| CT:SMI | 20 | No concerns | Some concerns | No concerns | No concerns | No concerns | Major concerns | Very low |
| CT:XMLI | 10 | No concerns | Some concerns | No concerns | Major concerns | No concerns | Major concerns | Very low |
| CT:YQFMI | 11 | No concerns | Some concerns | No concerns | No concerns | No concerns | Major concerns | Very low |
| ADR | Indirect evidence | | | | | | | |
| HQI:SFI | 0 | No concerns | Some concerns | No concerns | Major concerns | No concerns | Major concerns | Very low |
| HQI:SHMI | 0 | No concerns | Some concerns | No concerns | Major concerns | No concerns | Major concerns | Very low |
| HQI:SMI | 0 | No concerns | Some concerns | No concerns | Major concerns | No concerns | Major concerns | Very low |
| HQI:XMLI | 0 | No concerns | Some concerns | No concerns | Major concerns | No concerns | Major concerns | Very low |
| HQI:YQFMI | 0 | No concerns | Some concerns | No concerns | Major concerns | No concerns | Major concerns | Very low |
| SFI:SHMI | 0 | No concerns | Some concerns | No concerns | Major concerns | No concerns | Major concerns | Very low |
| SFI:SMI | 0 | No concerns | Some concerns | No concerns | Some concerns | Some concerns | Major concerns | Very low |
| SFI:XMLI | 0 | No concerns | Some concerns | No concerns | Major concerns | No concerns | Major concerns | Very low |
| SFI:YQFMI | 0 | No concerns | Some concerns | No concerns | Major concerns | No concerns | Major concerns | Very low |
| SHMI:SMI | 0 | No concerns | Some concerns | No concerns | Major concerns | No concerns | Major concerns | Very low |
| SHMI:XMLI | 0 | No concerns | Some concerns | No concerns | Major concerns | No concerns | Major concerns | Very low |
| SHMI:YQFMI | 0 | No concerns | Some concerns | No concerns | Major concerns | No concerns | Major concerns | Very low |
| SMI:XMLI | 0 | No concerns | Some concerns | No concerns | No concerns | No concerns | Major concerns | Very low |
| SMI:YQFMI | 0 | No concerns | Some concerns | No concerns | Major concerns | No concerns | Major concerns | Very low |
| XMLI:YQFMI | 0 | No concerns | Some concerns | No concerns | Some concerns | No concerns | Major concerns | Very low |
| BNP | Mixed evidence | | | | | | | |
| CT:HQI | 1 | No concerns | No concerns | No concerns | Major concerns | No concerns | Major concerns | Low |
| CT:SFI | 16 | No concerns | Some concerns | No concerns | No concerns | Major concerns | Major concerns | Very low |
| CT:SHMI | 2 | No concerns | No concerns | No concerns | No concerns | No concerns | Major concerns | Low |
| CT:SMI | 11 | No concerns | Some concerns | No concerns | No concerns | Major concerns | Major concerns | Very low |
| CT:XMLI | 13 | No concerns | Some concerns | No concerns | No concerns | Major concerns | Major concerns | Very low |
| CT:YQFMI | 1 | No concerns | Some concerns | No concerns | Major concerns | No concerns | Major concerns | Very low |
| BNP | Indirect evidence | | | | | | | |
| HQI:SFI | 0 | No concerns | Some concerns | No concerns | Major concerns | No concerns | Major concerns | Very low |
| HQI:SHMI | 0 | No concerns | No concerns | No concerns | No concerns | No concerns | Major concerns | Low |
| HQI:SMI | 0 | No concerns | Some concerns | No concerns | Some concerns | Some concerns | Major concerns | Very low |
| HQI:XMLI | 0 | No concerns | Some concerns | No concerns | Major concerns | No concerns | Major concerns | Very low |
| HQI:YQFMI | 0 | No concerns | Some concerns | No concerns | Major concerns | No concerns | Major concerns | Very low |
| SFI:SHMI | 0 | No concerns | Some concerns | No concerns | No concerns | Some concerns | Major concerns | Very low |
| SFI:SMI | 0 | No concerns | Some concerns | No concerns | Some concerns | Some concerns | Major concerns | Very low |
| SFI:XMLI | 0 | No concerns | Some concerns | No concerns | Major concerns | No concerns | Major concerns | Very low |
| SFI:YQFMI | 0 | No concerns | Some concerns | No concerns | Major concerns | No concerns | Major concerns | Very low |
| SHMI:SMI | 0 | No concerns | Some concerns | No concerns | No concerns | Major concerns | Major concerns | Very low |
| SHMI:XMLI | 0 | No concerns | Some concerns | No concerns | No concerns | Major concerns | Major concerns | Very low |
| SHMI:YQFMI | 0 | No concerns | Some concerns | No concerns | No concerns | No concerns | Major concerns | Very low |
| SMI:XMLI | 0 | No concerns | Some concerns | No concerns | Major concerns | No concerns | Major concerns | Very low |
| SMI:YQFMI | 0 | No concerns | Some concerns | No concerns | Major concerns | No concerns | Major concerns | Very low |
| XMLI:YQFMI | 0 | No concerns | Some concerns | No concerns | Major concerns | No concerns | Major concerns | Very low |
| NT-pro BNP | Mixed evidence | | | | | | | |
| CT:SFI | 8 | No concerns | No concerns | No concerns | No concerns | Major concerns | Major concerns | Low |
| CT:SHMI | 1 | No concerns | No concerns | No concerns | Major concerns | No concerns | Major concerns | Low |
| CT:SMI | 2 | No concerns | No concerns | No concerns | Major concerns | No concerns | Major concerns | Low |
| CT:XMLI | 8 | No concerns | No concerns | No concerns | No concerns | Major concerns | Major concerns | Low |
| CT:YQFMI | 16 | No concerns | Some concerns | No concerns | No concerns | Major concerns | Major concerns | Very low |
| NT-pro BNP | Indirect evidence | | | | | | | |
| SFI:SHMI | 0 | No concerns | No concerns | No concerns | Major concerns | No concerns | Major concerns | Low |
| SFI:SMI | 0 | No concerns | No concerns | No concerns | Major concerns | No concerns | Major concerns | Low |
| SFI:XMLI | 0 | No concerns | No concerns | No concerns | Some concerns | Some concerns | Major concerns | Low |
| SFI:YQFMI | 0 | No concerns | Some concerns | No concerns | Major concerns | No concerns | Major concerns | Very low |
| SHMI:SMI | 0 | No concerns | No concerns | No concerns | Major concerns | No concerns | Major concerns | Low |
| SHMI:XMLI | 0 | No concerns | No concerns | No concerns | Major concerns | No concerns | Major concerns | Low |
| SHMI:YQFMI | 0 | No concerns | Some concerns | No concerns | Major concerns | No concerns | Major concerns | Very low |
| SMI:XMLI | 0 | No concerns | No concerns | No concerns | Major concerns | No concerns | Major concerns | Low |
| SMI:YQFMI | 0 | No concerns | Some concerns | No concerns | Major concerns | No concerns | Major concerns | Very low |
| XMLI:YQFMI | 0 | No concerns | Some concerns | No concerns | Major concerns | No concerns | Major concerns | Very low |
| LVEF | Mixed evidence | | | | | | | |
| CT:HQI | 7 | No concerns | No concerns | No concerns | No concerns | Some concerns | Major concerns | Low |
| CT:SFI | 35 | No concerns | Some concerns | No concerns | No concerns | Some concerns | Major concerns | Very low |
| CT:SHMI | 14 | No concerns | No concerns | No concerns | No concerns | No concerns | Major concerns | Low |
| CT:SMI | 25 | No concerns | No concerns | No concerns | No concerns | No concerns | Major concerns | Low |
| CT:XMLI | 20 | No concerns | No concerns | No concerns | No concerns | Some concerns | Major concerns | Low |
| CT:YQFMI | 23 | No concerns | No concerns | No concerns | No concerns | Some concerns | Major concerns | Low |
| LVEF | Indirect evidence | | | | | | | |
| HQI:SFI | 0 | No concerns | Some concerns | No concerns | No concerns | Major concerns | Major concerns | Very low |
| HQI:SHMI | 0 | No concerns | No concerns | No concerns | No concerns | Some concerns | Major concerns | Low |
| HQI:SMI | 0 | No concerns | No concerns | No concerns | No concerns | Some concerns | Major concerns | Low |
| HQI:XMLI | 0 | No concerns | No concerns | No concerns | No concerns | Major concerns | Major concerns | Low |
| HQI:YQFMI | 0 | No concerns | No concerns | No concerns | No concerns | Major concerns | Major concerns | Low |
| SFI:SHMI | 0 | No concerns | Some concerns | No concerns | No concerns | Some concerns | Major concerns | Very low |
| SFI:SMI | 0 | No concerns | Some concerns | No concerns | No concerns | Some concerns | Major concerns | Very low |
| SFI:XMLI | 0 | No concerns | Some concerns | No concerns | No concerns | Major concerns | Major concerns | Very low |
| SFI:YQFMI | 0 | No concerns | Some concerns | No concerns | No concerns | Some concerns | Major concerns | Very low |
| SHMI:SMI | 0 | No concerns | No concerns | No concerns | No concerns | Some concerns | Major concerns | Low |
| SHMI:XMLI | 0 | No concerns | No concerns | No concerns | No concerns | Some concerns | Major concerns | Low |
| SHMI:YQFMI | 0 | No concerns | No concerns | No concerns | No concerns | Some concerns | Major concerns | Low |
| SMI:XMLI | 0 | No concerns | No concerns | No concerns | No concerns | Some concerns | Major concerns | Low |
| SMI:YQFMI | 0 | No concerns | No concerns | No concerns | No concerns | Some concerns | Major concerns | Low |
| XMLI:YQFMI | 0 | No concerns | No concerns | No concerns | No concerns | Some concerns | Major concerns | Low |
| LVED | Mixed evidence | | | | | | | |
| CT:HQI | 1 | No concerns | No concerns | No concerns | No concerns | Major concerns | Major concerns | Low |
| CT:SFI | 8 | No concerns | Some concerns | No concerns | No concerns | Major concerns | Major concerns | Very low |
| CT:SHMI | 7 | No concerns | Some concerns | No concerns | No concerns | Major concerns | Major concerns | Very low |
| CT:SMI | 10 | No concerns | Some concerns | No concerns | No concerns | Major concerns | Major concerns | Very low |
| CT:XMLI | 5 | No concerns | No concerns | No concerns | No concerns | Major concerns | Major concerns | Low |
| CT:YQFMI | 14 | No concerns | Some concerns | No concerns | No concerns | Major concerns | Major concerns | Very low |
| LVED | Indirect evidence | | | | | | | |
| HQI:SFI | 0 | No concerns | Some concerns | No concerns | No concerns | Major concerns | Major concerns | Very low |
| HQI:SHMI | 0 | No concerns | Some concerns | No concerns | No concerns | Major concerns | Major concerns | Very low |
| HQI:SMI | 0 | No concerns | Some concerns | No concerns | No concerns | Major concerns | Major concerns | Very low |
| HQI:XMLI | 0 | No concerns | No concerns | No concerns | No concerns | Major concerns | Major concerns | Low |
| HQI:YQFMI | 0 | No concerns | Some concerns | No concerns | No concerns | Major concerns | Major concerns | Very low |
| SFI:SHMI | 0 | No concerns | Some concerns | No concerns | No concerns | Major concerns | Major concerns | Very low |
| SFI:SMI | 0 | No concerns | Some concerns | No concerns | No concerns | Major concerns | Major concerns | Very low |
| SFI:XMLI | 0 | No concerns | Some concerns | No concerns | No concerns | Major concerns | Major concerns | Very low |
| SFI:YQFMI | 0 | No concerns | Some concerns | No concerns | No concerns | Major concerns | Major concerns | Very low |
| SHMI:SMI | 0 | No concerns | Some concerns | No concerns | No concerns | Major concerns | Major concerns | Very low |
| SHMI:XMLI | 0 | No concerns | Some concerns | No concerns | No concerns | Major concerns | Major concerns | Very low |
| SHMI:YQFMI | 0 | No concerns | Some concerns | No concerns | No concerns | Major concerns | Major concerns | Very low |
| SMI:XMLI | 0 | No concerns | Some concerns | No concerns | No concerns | Major concerns | Major concerns | Very low |
| SMI:YQFMI | 0 | No concerns | Some concerns | No concerns | No concerns | Major concerns | Major concerns | Very low |
| XMLI:YQFMI | 0 | No concerns | Some concerns | No concerns | No concerns | Major concerns | Major concerns | Very low |
| 6MWT | Mixed evidence | | | | | | | |
| CT:HQI | 1 | No concerns | Some concerns | No concerns | No concerns | Major concerns | Major concerns | Very low |
| CT:SFI | 3 | No concerns | Some concerns | No concerns | No concerns | No concerns | Major concerns | Very low |
| CT:SMI | 5 | No concerns | Some concerns | No concerns | No concerns | Some concerns | Major concerns | Very low |
| CT:XMLI | 4 | No concerns | Some concerns | No concerns | No concerns | Some concerns | Major concerns | Very low |
| CT:YQFMI | 4 | No concerns | Some concerns | No concerns | No concerns | Some concerns | Major concerns | Very low |
| 6MWT | Indirect evidence | | | | | | | |
| HQI:SFI | 0 | No concerns | Some concerns | No concerns | No concerns | Some concerns | Major concerns | Very low |
| HQI:SMI | 0 | No concerns | Some concerns | No concerns | No concerns | Major concerns | Major concerns | Very low |
| HQI:XMLI | 0 | No concerns | Some concerns | No concerns | No concerns | Major concerns | Major concerns | Very low |
| HQI:YQFMI | 0 | No concerns | Some concerns | No concerns | Some concerns | Some concerns | Major concerns | Very low |
| SFI:SMI | 0 | No concerns | Some concerns | No concerns | No concerns | Some concerns | Major concerns | Very low |
| SFI:XMLI | 0 | No concerns | Some concerns | No concerns | No concerns | Some concerns | Major concerns | Very low |
| SFI:YQFMI | 0 | No concerns | Some concerns | No concerns | No concerns | Some concerns | Major concerns | Very low |
| SMI:XMLI | 0 | No concerns | Some concerns | No concerns | No concerns | Major concerns | Major concerns | Very low |
| SMI:YQFMI | 0 | No concerns | Some concerns | No concerns | No concerns | Major concerns | Major concerns | Very low |
| XMLI:YQFMI | 0 | No concerns | Some concerns | No concerns | No concerns | Major concerns | Major concerns | Very low |

**Supplementary Table S8.** Overall and subgroup meta-analysis results.

**Table 8.1 Overall analyses by intervention**

Table 8.1.1 Overall analyses by intervention of CER

| Interventions | n | I²(%) | OR (95%CI) |
| --- | --- | --- | --- |
| SFI+CT vs CT | 25 | 0.00 | 3.30(2.54, 4.29) |
| SMI+CT vs CT | 23 | 0.00 | 4.48(3.48, 5.76) |
| SHMI+CT vs CT | 15 | 0.00 | 4.12(3.00, 5.79) |
| HQI+CT vs CT | 7 | 0.00 | 3.27(2.09, 5.11) |
| XMLI+CT vs CT | 17 | 0.00 | 4.30(3.08, 6.00) |
| YQFMI+CT vs CT | 15 | 0.00 | 5.00(3.43, 7.28) |
| Overall | 103 | 0.00 | 4.06(3.57, 4.61) |

Table 8.1.2 Overall analyses by intervention of ADR

| Interventions | n | I²(%) | OR (95%CI) |
| --- | --- | --- | --- |
| SFI+CT vs CT | 6 | 23.40 | 1.97(1.07, 3.62) |
| SMI+CT vs CT | 17 | 0.00 | 2.65(1.96, 3.58) |
| SHMI+CT vs CT | 2 | 49.20 | 1.26(0.46, 3.49) |
| HQI+CT vs CT | 2 | 0.00 | 1.76(0.24, 12.85) |
| XMLI+CT vs CT | 9 | 17.80 | 1.29(0.83, 2.03) |
| YQFMI+CT vs CT | 10 | 8.40 | 2.14(1.41, 3.23) |
| Overall | 65 | 9.30 | 2.08(1.71, 2.53) |

Table 8.1.3 Overall analyses by intervention of BNP

| Interventions | n | I²(%) | SMD (95%CI) |
| --- | --- | --- | --- |
| SFI+CT vs CT | 16 | 97.40 | -1.90(-2.76, -1.39) |
| SMI+CT vs CT | 11 | 97.00 | -2.79(-3.68, -1.91) |
| SHMI+CT vs CT | 2 | 96.20 | -5.45(-9.35, -1.55) |
| HQI+CT vs CT | 1 | 0.00 | 0.07(-0.31, -0.45) |
| XMLI+CT vs CT | 12 | 93.70 | -1.70(-2.26, -1.14) |
| YQFMI+CT vs CT | 1 | 0.00 | -0.25(-0.80, -0.31) |
| Overall | 44 | 97.00 | -2.17(-2.63, -1.72) |

Table 8.1.4 Overall analyses by intervention of NT-pro BNP

| Interventions | n | I²(%) | SMD (95%CI) |
| --- | --- | --- | --- |
| SFI+CT vs CT | 8 | 92.40 | -1.30(-1.96, 0.63) |
| SMI+CT vs CT | 2 | 75.10 | -0.68(-1.28, -0.07) |
| SHMI+CT vs CT | 1 | 0.00 | -2.83(-3.44, -2.21) |
| XMLI+CT vs CT | 7 | 96.50 | -2.94(-4.04, -1.84) |
| YQFMI+CT vs CT | 16 | 98.30 | -1.81(-2.88, -0.74) |
| Overall | 35 | 97.60 | -1.97(-2.56, -1.38) |

Table 8.1.5 Overall analyses by intervention of LVEF

| Interventions | n | I²(%) | MD (95%CI) |
| --- | --- | --- | --- |
| SFI+CT vs CT | 35 | 98.50 | 5.43(4.16, 6.70) |
| SMI+CT vs CT | 24 | 99.40 | 9.61(6.78, 12.43) |
| SHMI+CT vs CT | 14 | 99.90 | 7.35(5.89, 8.81) |
| HQI+CT vs CT | 7 | 81.10 | 4.94(3.48, 6.40) |
| XMLI+CT vs CT | 20 | 98.80 | 5.86(3.79, 7.92) |
| YQFMI+CT vs CT | 23 | 99.40 | 3.74(1.59, 5.90) |
| Overall | 124 | 97.60 | 6.23(5.65, 6.80) |

Table 8.1.6 Overall analyses by intervention of LVED

| Interventions | n | I²(%) | MD (95%CI) |
| --- | --- | --- | --- |
| SFI+CT vs CT | 8 | 97.60 | -2.69(-7.1, 1.75) |
| SMI+CT vs CT | 10 | 94.40 | -6.37(-8.19, -4.54) |
| SHMI+CT vs CT | 7 | 70.90 | -3.12(-4.56, -1.67) |
| HQI+CT vs CT | 1 | 0.00 | -6.11(-7.29, -4.93) |
| XMLI+CT vs CT | 5 | 90.40 | -4.92(-6.39, -3.45) |
| YQFMI+CT vs CT | 14 | 99.90 | -8.74(-16.74, -0.74) |
| Overall | 45 | 99.80 | -5.79(-9.00, -2.58) |

Table 8.1.7 Overall analyses by intervention of 6MWT

| Interventions | n | I²(%) | MD (95%CI) |
| --- | --- | --- | --- |
| SFI+CT vs CT | 3 | 97.80 | 99.26(29.01, 169.51) |
| SMI+CT vs CT | 5 | 94.60 | 49.18(30.76, 67.60) |
| HQI+CT vs CT | 1 | 0.00 | 42.11(21.86, 62.36) |
| XMLI+CT vs CT | 4 | 90.90 | 42.46(20.71, 62.21) |
| YQFMI+CT vs CT | 4 | 0.00 | 50.45(42.08, 58.82) |
| Overall | 17 | 94.40 | 57.11(45.89, 68.34) |

**Table 8.2 Subgroup analyses**

Table 8.2.1 Subgroup analysis of BNP

| Grouping criteria | Subgroups | n | I²(%) | SMD (95%CI) |
| --- | --- | --- | --- | --- |
| Mean age | < 65 years | 27 | 97.20 | -2.94(-3.60, -2.29) |
|  | ≥ 65 years | 17 | 95.10 | -1.09(-1.61, -0.57) |
| Treatment course | ≤ 14 days | 38 | 96.90 | -2.09(-2.58, -1.59) |
|  | > 14 days | 6 | 96.40 | -2.69(-3.79, -1.59) |
| Dose | low | 19 | 97.30 | -2.13(-2.86, -1.40) |
|  | high | 25 | 96.70 | -2.20(-2.79, -1.60) |
| Classification | acute | 12 | 97.60 | -1.52(-2.47, -0.57) |
|  | chronic | 32 | 96.60 | -2.41(-2.63, -1.90) |

Table 8.2.2 Subgroup analysis of NT-pro BNP

| Grouping criteria | Subgroups | n | I²(%) | SMD (95%CI) |
| --- | --- | --- | --- | --- |
| Mean age | < 65 years | 17 | 96.40 | -1.61(-2.25, -0.96) |
|  | ≥ 65 years | 18 | 98.20 | -2.24(-3.27, -1.22) |
| Treatment course | ≤ 14 days | 30 | 97.60 | -1.88(-2.54, -1.23) |
|  | > 14 days | 5 | 96.60 | -1.94(-3.15, -0.73) |
| Dose | low | 11 | 92.00 | -1.01(-1.55, -0.47) |
|  | high | 24 | 98.10 | -2.39(-3.23, -1.55) |
| Classification | acute | 29 | 83.60 | -3.37(-4.04, -2.70) |
|  | chronic | 6 | 97.50 | -1.67(-2.28, -1.05) |

Table 8.2.3 Subgroup analysis of LVEF

| Grouping criteria | Subgroups | n | I²(%) | MD (95%CI) |
| --- | --- | --- | --- | --- |
| Mean age | < 65 years | 81 | 98.90 | 7.27(6.48, 8.05) |
|  | ≥ 65 years | 43 | 99.80 | 4.29(2.56, 6.02) |
| Treatment course | ≤ 14 days | 97 | 99.70 | 6.20(5.55, 6.86) |
|  | > 14 days | 27 | 99.10 | 6.33(4.65, 8.01) |
| Dose | low | 57 | 99.60 | 6.57(5.21, 7.93) |
|  | high | 67 | 99.50 | 6.04(5.01, 7.07) |
| Baseline LVEF | < 40% | 81 | 99.70 | 6.46(5.79, 7.12) |
|  | ≥ 40% | 43 | 99.50 | 5.78(3.66, 7.90) |
| Classification | acute | 27 | 95.90 | 6.91(5.48, 8.34) |
|  | chronic | 97 | 99.70 | 6.06(5.41, 6.70) |

Table 8.2.4 Subgroup analysis of LVEDD

| Grouping criteria | Subgroups | n | I²(%) | MD (95%CI) |
| --- | --- | --- | --- | --- |
| Mean age | < 65 years | 32 | 99.80 | -6.81(-10.92, -2.71) |
|  | ≥ 65 years | 13 | 96.10 | -3.33(5.43, -1.24) |
| Treatment course | ≤ 14 days | 36 | 99.80 | -5.86(-9.69, -2.03) |
|  | > 14 days | 9 | 92.50 | -5.53(-7.29, -3.77) |
| Dose | low | 23 | 90.40 | -4.82(-5.87, -3.77) |
|  | high | 22 | 99.90 | -6.95(-12.30, -1.60) |
| Classification | acute | 8 | 96.70 | -2.98(-5.71, -0.25) |
|  | chronic | 37 | 99.80 | -6.40(-10.13, -2.68) |

Table 8.2.5 Subgroup analysis of 6MWT

| Grouping criteria | Subgroups | n | I²(%) | MD (95%CI) |
| --- | --- | --- | --- | --- |
| Mean age | < 65 years | 10 | 90.10 | 52.46(41.76, 63.15) |
|  | ≥ 65 years | 7 | 96.90 | 64.42(29.78, 99.06) |
| Treatment course | ≤ 14 days | 14 | 95.40 | 59.16(41.87, 76.45) |
|  | > 14 days | 3 | 46.20 | 48.07(37.46, 58.68) |
| Dose | low | 6 | 93.10 | 57.11(45.89, 68.34) |
|  | high | 11 | 95.20 | 50.45(42.08, 58.82) |

# 2 Supplementary Figure

**Supplementary Figure S1.** League tables from the sensitivity analyses. Values are effect estimates with 95% confidence intervals. ADR, adverse drug reaction; BNP, B-type natriuretic peptide; CT, conventional treatment; HQI, Huangqi injection; LVEF, left ventricular ejection fraction; LVED, left ventricular end-diastolic dimension; NT-proBNP, N-terminal pro-B-type natriuretic peptide; SFI, Shenfu injection; SHMI, Shengmai injection; SMI, Shenmai injection; XMLI, Xinmailong injection; YQFMI, Yiqifumai injection; 6MWT, 6-minute walk test.
